# Insulin Resistance and Risk of Incident Cardiovascular Events in Adults without Diabetes: Meta-Analysis

**DOI:** 10.1371/journal.pone.0052036

**Published:** 2012-12-28

**Authors:** Karin B. Gast, Nathanja Tjeerdema, Theo Stijnen, Johannes W. A. Smit, Olaf M. Dekkers

**Affiliations:** 1 Department of Internal Medicine, Leiden University Medical Center, Leiden, The Netherlands; 2 Department of Clinical Epidemiology, Leiden University Medical Center, Leiden, The Netherlands; 3 Department of Medical Statistics and Bioinformatics, Leiden University Medical Center, Leiden, The Netherlands; 4 Department of Endocrinology, Leiden University Medical Center, Leiden, The Netherlands; Universidad Peruana de Ciencias Aplicadas (UPC), Peru

## Abstract

**Background:**

Glucose, insulin and Homeostasis Model Assessment Insulin Resistance (HOMA-IR) are markers of insulin resistance. The objective of this study is to compare fasting glucose, fasting insulin concentrations and HOMA-IR in strength of association with incident cardiovascular disease.

**Methods:**

We searched the PubMed, MEDLINE, EMBASE, Web of Science, ScienceDirect and Cochrane Library databases from inception to March, 2011, and screened reference lists. Cohort studies or nested case-control studies that investigated the association between fasting glucose, fasting insulin or HOMA-IR and incident cardiovascular disease, were eligible. Two investigators independently performed the article selection, data extraction and risk of bias assessment. Cardiovascular endpoints were coronary heart disease (CHD), stroke or combined cardiovascular disease. We used fixed and random-effect meta-analyses to calculate the pooled relative risk for CHD, stroke and combined cardiovascular disease, comparing high to low concentrations of glucose, insulin or HOMA-IR. Study heterogeneity was calculated with the I^2^ statistic. To enable a comparison between cardiovascular disease risks for glucose, insulin and HOMA-IR, we calculated pooled relative risks per increase of one standard deviation.

**Results:**

We included 65 studies (involving 516,325 participants) in this meta-analysis. In a random-effect meta-analysis the pooled relative risk of CHD (95% CI; I^2^) comparing high to low concentrations was 1.52 (1.31, 1.76; 62.4%) for glucose, 1.12 (0.92, 1.37; 41.0%) for insulin and 1.64 (1.35, 2.00; 0%) for HOMA-IR. The pooled relative risk of CHD per one standard deviation increase was 1.21 (1.13, 1.30; 64.9%) for glucose, 1.04 (0.96, 1.12; 43.0%) for insulin and 1.46 (1.26, 1.69; 0.0%) for HOMA-IR.

**Conclusions:**

The relative risk of cardiovascular disease was higher for an increase of one standard deviation in HOMA-IR compared to an increase of one standard deviation in fasting glucose or fasting insulin concentration. It may be useful to add HOMA-IR to a cardiovascular risk prediction model.

## Introduction

Cardiovascular disease is worldwide the leading cause of death [1]. Type 2 diabetes contributes importantly to cardiovascular disease, because it is highly prevalent and doubles cardiovascular disease risk [2], [3]. Before type 2 diabetes is diagnosed, insulin resistance can be present for years, thereby increasing insulin and glucose concentrations [4], [5].

Recent meta-analyses have shown that elevated insulin and glucose concentrations in persons without diabetes were associated with an increased cardiovascular disease risk [3], [6]. In accordance, mechanistic studies have shown that elevated glucose and insulin concentrations can be pro-atherogenic [7], [8]. Elevated insulin and glucose concentrations are direct consequences of insulin resistance. Insulin resistance can promote the development of atherosclerosis through elevated glucose and insulin concentrations, but also through mechanisms that involve dyslipidemia, hypertension, and inflammation [7], [9]. Therefore, cardiovascular disease may be caused by insulin resistance rather than being a consequence of the toxic effects of elevated insulin or glucose concentrations. A validated and frequently used marker of insulin resistance is the Homeostasis Model Assessment Insulin Resistance (HOMA-IR). Since, HOMA-IR incorporates both glucose and insulin concentrations and represents insulin resistance, which can promote atherosclerosis trough several mechanisms [7], [9], it might be more strongly associated with cardiovascular disease than individual glucose or insulin concentrations. No meta-analysis thus far, has compared the strength of association between HOMA-IR and cardiovascular disease to associations between fasting glucose, fasting insulin and cardiovascular disease.

Our aim was to perform a systematic review and meta-analysis on the association between fasting glucose, fasting insulin, HOMA-IR and incident cardiovascular disease in individuals without diabetes. Our second aim was to compare fasting glucose, fasting insulin and HOMA-IR in strength of association with incident cardiovascular disease. We hypothesized that HOMA-IR is more strongly associated with incident cardiovascular disease than fasting glucose or fasting insulin.

## Methods

### Data Sources and Searches

We searched the following databases from their inception to February 23, 2010: PubMed, MEDLINE, EMBASE, Web of Science, ScienceDirect and Cochrane Library. We updated the search to February 29th, 2011 for the MEDLINE and PubMed databases. The search strategy was optimized for all consulted databases, taking into account the differences of the various controlled vocabularies as well as the differences of database-specific technical variations (e.g. the use of quotation marks). The reference lists of all potentially relevant articles were screened for additional publications. Detailed and database specific information about the search strategy is shown in Table S1.

### Study Selection

The aim of our meta-analysis was to investigate the association between fasting glucose, fasting insulin, HOMA-IR and incident cardiovascular disease in individuals without diabetes at baseline. Cohort studies that measured glucose, insulin or HOMA-IR and reported original data on their association with cardiovascular disease, were eligible. We considered only cohort studies or nested case-control studies that measured glucose or insulin concentrations prior to the assessment of cardiovascular disease with a subsequent follow-up of minimally one year. No cross-sectional studies were eligible. In addition, articles in other languages than English were not eligible.

Since anti-diabetic drugs influence insulin and glucose concentrations, study populations should preferably have excluded participants with overt diabetes at baseline. However, population based studies that did not exclude participants with overt diabetes at baseline were eligible for inclusion. We excluded studies performed in populations exclusively consisting of persons with known diabetes or cohorts restricted to specific populations such as intensive care or transplant patients.

Studies that measured glucose or insulin concentrations in the fasting state were eligible for inclusion. Unfortunately, no uniform definition of fasting exists and many different definitions are being used [10]. Concentrations were considered to be fasting if study participants abstained from food for at least eight hours. Studies that reported the glucose or insulin concentrations to be fasting or measured after an overnight fast, but did not report the time span of fasting, were not excluded.

Studies reporting on at least one of the following endpoints were eligible: myocardial infarction, angina pectoris, stroke (ischemic or hemorrhagic), arrhythmias, congestive heart failure or sudden cardiac death separately or combinations. Studies that combined these endpoints with peripheral arterial disease, arterial aneurysm or arterial dissection in a composite endpoint were not excluded.

Furthermore, to be included studies should (1) report the association by comparing categories (percentiles or cut-off values), (2) express the association as relative risks (hazard ratios, rate ratios, risk ratios or odds ratios) with corresponding standard errors, confidence intervals or exact p-values and (3) adjust effect estimates at least for age and sex. In case of multiple publications arising from the same study population we included the study with the highest number of participants or the longest follow-up.

### Data Extraction and Quality Assessment

Two investigators (K.G. and N.T.) independently performed the article selection based on titles and abstracts, data extraction and risk of bias assessment using a standard data sheet. Disagreement was resolved by consensus or by a third party (O.D.).

If necessary, glucose and insulin concentrations were recalculated to the international system of units (i.e. mmol/L for glucose and pmol/L for insulin) [11]. Values for HOMA-IR were based on values provided by the authors of included studies. In general, HOMA-IR is calculated by the formula: (fasting insulin x fasting glucose)/22.5 or by the more recently updated computer model [12]. We recalculated HOMA-IR values for studies that reported HOMA insulin sensitivity, which is the reciprocal of HOMA-IR.

We categorized study endpoints as (fatal or non-fatal): (1) coronary heart disease (CHD), (2) stroke and as (3) combined cardiovascular disease outcome (CVD), including studies contributing to 1 or 2. CHD was defined as myocardial infarction or angina pectoris; stroke consisted of hemorrhagic or ischemic stroke and CVD consisted of myocardial infarction, angina pectoris, hemorrhagic stroke, ischemic stroke, arrhythmias, congestive heart failure or sudden cardiac death.

Risk of bias assessment was based on design elements of cohort studies and nested case-control studies that could potentially bias the association between fasting glucose, fasting insulin, HOMA-IR and cardiovascular disease. Potential sources of bias were assessed by using a predefined assessment form. Dimensions considered for both cohort and nested case-control studies were (1) presence of overt diabetes at baseline, (2) presence of cardiovascular disease at baseline, (3) adequacy of exposure measurement, (4) missing glucose, insulin or HOMA-IR data, (5) adequacy of endpoint ascertainment. Bias was considered to be likely present when: (1) study populations had overt diabetes prevalence of twice their country specific diabetes prevalence estimates of 2011 [13]; indicating that studies have selected their study population based on high glucose concentrations (selection bias), (2) persons with prevalent cardiovascular disease according to their outcome definition were not excluded; (3) the time span of fasting was not reported, (4) ≥10% missing data of the exposure except when data was missing completely at random (e.g. in the case of later introduction of the measurement), (5) outcome classification was based on self- or family reports, (6) there was ≥10% loss to follow-up. Reliable methods of outcome assessment were assessment by medical records, death certificates or hospital discharge records. Diagnosis of myocardial infarction was considered reliable when WHO MONICA criteria or Minnesota coding of electrocardiograms during follow-up visits were used [14]–[16].

### Data Synthesis and Analysis

Hazard ratios, rate ratios, risk ratios or odds ratios (relative risks) of cardiovascular disease comparing high to low concentrations of glucose, insulin or HOMA-IR values were extracted. If necessary, we recalculated these relative risks in a way that the lowest category (percentile or cut-off value) comprised the reference category. Our first aim was to estimate the pooled relative risk for cardiovascular disease, when comparing categories (based on either percentiles or cut-offs) of high concentrations of glucose, insulin or HOMA-IR to categories of lower concentrations. We pooled maximally adjusted effect measures of studies with corresponding 95% confidence intervals (CI). For all analyses, both a fixed and a random-effect meta-analysis were performed. Study heterogeneity was calculated with the I^2^ statistic. Elements of the risk of bias assessment were used to explore potential heterogeneity in sensitivity analyses. We assessed the presence of funnel plot asymmetry by calculating Egger’s test [17].

Our second aim was to compare fasting glucose, fasting insulin and HOMA-IR in strength of association with cardiovascular disease by comparing pooled standardized relative risks (i.e. risk increase per increase of one standard deviation). First, we calculated the standard deviation per exposure by pooling reported standard deviations with a weight factor based on study size. Secondly, we applied the method of Hartemink et al. [18] to calculate an overall relative risk per one unit increase of the exposure. Then, we multiplied the logarithm of the relative risks by the pooled standard deviation of the exposure. In short, the method of Hartemink et al. [18] assumes a log-linear relation between the risk and the exposure. The input of the algorithm consists of the means and variances of the exposure within each category of the exposure, the log relative risks of the categories with respect to a reference category, and the number of cases within each category. To determine the category means and variances we applied various methods, depending on the kind of data reported in the article. We assumed a lognormal distribution for the exposures. Finally, we tested differences in pooled relative risks between the three exposures by using multivariate meta-analysis. Relative risks obtained from the same study (i.e. for studies that reported relative risks for more than one exposure) are likely to be correlated and this correlation is taken into account by multivariate meta-analysis.

We investigated sex differences in studies that presented sex-specific relative risks of cardiovascular disease by performing meta-analyses stratified by sex. Statistical analyses were performed with STATA Statistical Software *(Statacorp, College Station, Texas, USA)*, version 11.2 and SAS software (*SAS Institute Inc., Cary, NC, USA*), version 9.2.

## Results

### Search Results

**Figure 1:**
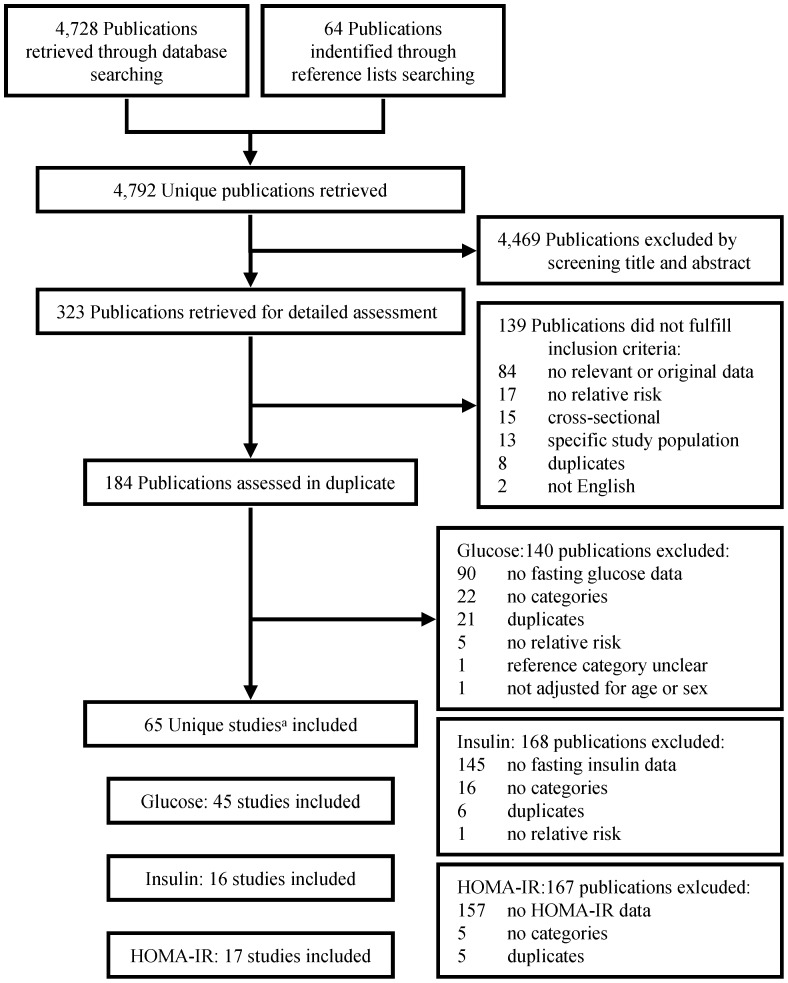
Summary of search results. ^a^One publication consisted of two studies. HOMA-IR, Homeostasis Model Assessment insulin resistance.

We identified 4,792 unique publications by database search (MEDLINE: n = 2,095, PubMed: n = 1,480, EMBASE n = 852, Cochrane: n = 112, ScienceDirect: n = 103, Web of Science: n = 86) and by screening reference lists of potentially relevant articles (n = 64). After exclusion of 4,469 publications by screening title and abstract, 323 publications were retrieved for detailed assessment of which 184 fulfilled inclusion criteria and were assessed in duplicate. To avoid multiple inclusions of the same study participants, we excluded 32 publications originating from the same study populations and included the publication with the largest population or the longest follow-up. Sixty-five studies (from 64 publications) were included. Forty-five studies presented data on fasting glucose, 17 studies presented data on fasting insulin and 16 studies presented data on HOMA-IR (Figure 1).

### Study Characteristics

Study characteristics of the included studies are summarized in table 1. Sixty-four cohort studies and 1 nested case-control study were included. The controls in this case-cohort study were matched on time and therefore the odds ratio corresponds to a rate ratio [19]. Fifty-six studies presented a hazard ratio and nine studies presented an odds ratio. Most study populations consisted of both men and women. Individual study characteristics of included studies are shown in Table S2.

**Table 1:** Study characteristics of the included studies summarized for three exposures.

	Exposure		
Characteristic	Glucose(45 studies)	Insulin(16 studies)	HOMA-IR(17 studies)
Total participants	450,487	46,236	51,161
Participants per study (range)	541–63,443	541–13,446	839–6,942
Year of publication	1983–2010	1992–2010	2001–2010
Mean follow-up (years, range)	3.2–23.5	5.0–22.3^a^	2.2–30
Study design			
Cohort	45	15	17
Nested case-control	0	1	0
CHD endpoint			
Number of studies	23	9	7
Events per study	23–4,490^b^	16–677	33–169^b^
Total events	10,884^b^	2,149	441^b^
Stroke endpoint			
Number of studies	14	2	4
Events per study	13–405^c^	25–70	23–70^b^
Total events	1,936^c^	95	164^b^
Combined CVD endpoint			
Number of studies	45	16	17
Events per study	23–4,490^b^	16–492	58–340
Total events	19,993^b^	3,329	3,035

Data are presented as number or range.

a Three studies did not report follow-up time.

b Two studies did not report the number of participants who encountered the outcome of interest.

c One study did not report the number of participants who encountered the outcome of interest.

HOMA-IR, Homeostasis Model Assessment Insulin Resistance; CHD, coronary heart disease; CVD, cardiovascular disease.

### Risk of Bias

The risk of bias assessment is summarized in Table S3 and shown per study in Table S4. Most studies excluded persons with overt diabetes at baseline. One study included persons with prevalent cardiovascular disease and this was unclear in 20 studies. Twenty-two studies did not specify the time span of fasting or whether participants had an overnight fast. Five studies had more than 10% missing data for glucose, four studies for insulin and three studies for HOMA-IR which was not reported to be completely at random. In 13 studies we considered bias to be likely present due to inadequate outcome assessment. The percentage of participants that were loss to follow-up ranged from 0% to 42%. Seven studies had a loss to follow-up of more than 10% and this was unclear in most studies. The p-values of Egger’s test were 0.08 for glucose, <0.01 for insulin and <0.01 for HOMA-IR.

### Comparison between Glucose, Insulin and HOMA-IR

**Figure 2:**
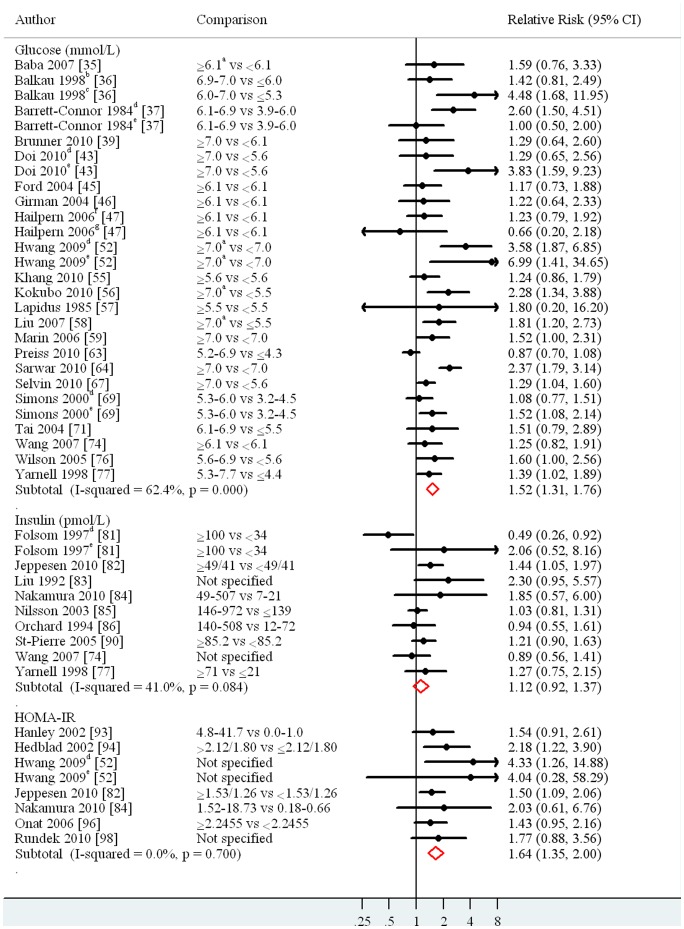
Random-effect meta-analyses of coronary heart disease risk for the highest category of glucose, insulin or HOMA-IR compared to the lowest category. ^a^Or known diabetes was used to define the highest category. ^b^Paris Prospective Study. ^c^Helsinki Policemen Study. ^d^Men. ^e^Women. ^f^Glomerular Filtration Rate ≥60 ml/min/1.73 m^2. g^Glomerular Filtration Rate <60 ml/min/1.73 m^2.^ References are listed in References S1. 95% CI, 95% confidence interval; vs, versus; I-squared, measure of heterogeneity; HOMA-IR, Homeostasis Model Assessment Insulin Resistance.

In a random-effect meta-analysis the pooled relative risk of CHD comparing the highest versus the lowest category was 1.52 (95% CI: 1.31, 1.76; I^2^∶62.4%) for glucose, 1.12 (95% CI: 0.92, 1.37; I^2^∶41.0%) for insulin and 1.64 (95% CI: 1.35, 2.00; I^2^∶0%) for HOMA-IR (Figure 2 and Figure S1). The pooled relative risks for the association with stroke and CVD, and meta-analyses stratified by sex for studies that provided sex-specific relative risks are summarized in Figure S1.

**Figure 3:**
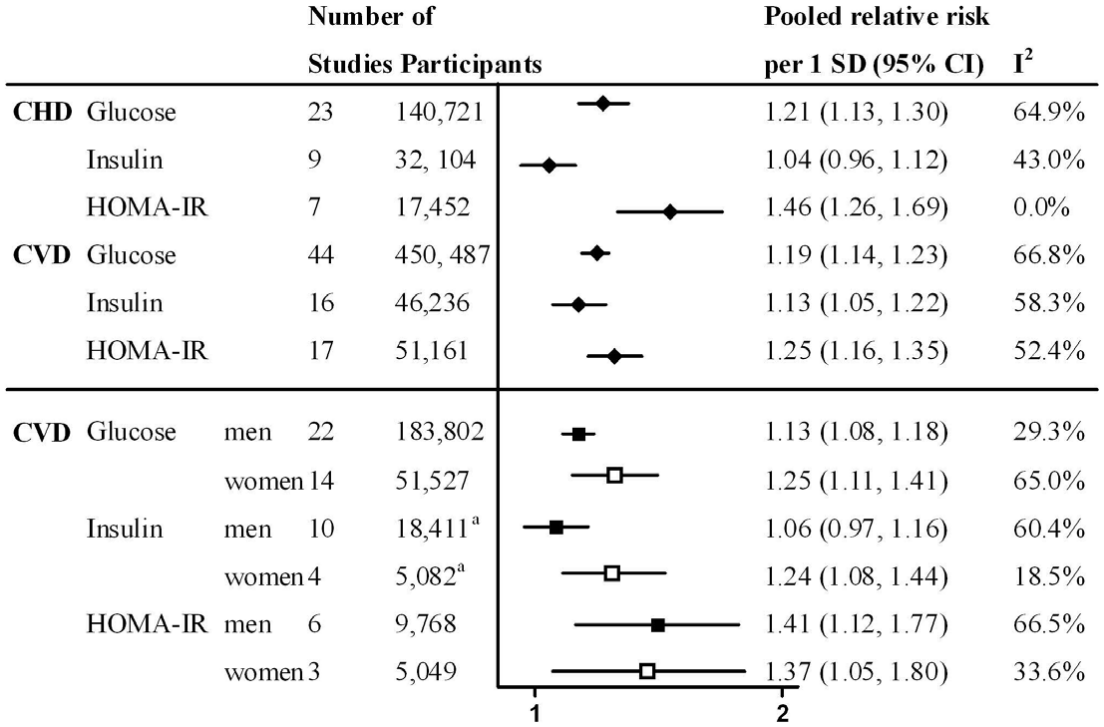
Results of random-effect meta-analyses comparing cardiovascular disease risk for an increase of one standard deviation. ^a^1 study did not specify sex-specific numbers. SD, standard deviation; 95% CI, 95% confidence interval; I^2^, measure of heterogeneity; CHD, coronary heart disease and is defined as fatal or non-fatal myocardial infarction or angina pectoris; CVD, cardiovascular disease and is defined as myocardial infarction, angina pectoris, hemorrhagic stroke, ischemic stroke, arrhythmias, congestive heart failure or sudden cardiac death; HOMA-IR, Homeostasis Model Assessment Insulin Resistance.

To enable a direct comparison between CHD and CVD risks for glucose, insulin and HOMA-IR we calculated pooled relative risks for an increase of one standard deviation [18]. We did not investigate the endpoint stroke, because only two studies investigated the association between insulin and stroke. The relative risks per increase of one standard deviation for glucose (1.05 mmol/L), insulin (43.53 pmol//L) and HOMA-IR (2.23 units) are shown in Figure 3. The pooled relative risk of CHD per one standard deviation increase was 1.21 (95% CI: 1.13, 1.30; I^2^∶64.9%) for glucose, 1.04 (95% CI: 0.96, 1.12; I^2^∶43.0%) for insulin and 1.46 (95% CI: 1.26, 1.69; I^2^∶0.0%) for HOMA-IR. The pooled relative risks of CHD for glucose, insulin, and HOMA-IR were all statistically different from each other (p-values: <0.05). The pooled relative risks of CVD for glucose, insulin, and HOMA-IR were not statistically different (p-value: 0.27).

Thirty-three studies provided sex-specific relative risks of CVD. Few studies provided relative risks of CHD or stroke for women and therefore we only investigated sex differences for incident CVD. Women had higher relative risks of CVD per one standard deviation increase of glucose (1.25 (95% CI: 1.11, 1.41; I^2^∶65.0%) versus 1.13 (95% CI: 1.08, 1.18; I^2^∶29.3%); p-value: 0.01) and insulin (1.24 (95% CI: 1.08, 1.44; I^2^∶18.5%) versus 1.06 (95% CI: 0.97, 1.16; I^2^∶60.4%); p-value: 0.03) and lower relative risk of CVD per one standard deviation increase of HOMA-IR (1.37 (95% CI: 1.05, 1.80; I^2^ 33.6%) versus 1.41 (95% CI: 1.12, 1.77; I^2^ 66.5%); p-value: 0.73) (Figure 3). In sensitivity analyses we excluded studies which had a high risk of bias based on items of the risk of bias assessment. The results of the meta-analyses were materially unchanged.

## Discussion

The present meta-analyses showed that fasting glucose, fasting insulin and HOMA-IR were all associated with incident cardiovascular disease in individuals without diabetes. In a standardized meta-analysis we found that coronary heart disease risk increased with 46% for an increase of one standard deviation in HOMA-IR concentration compared to an increase of 21% for fasting glucose concentration and an increase of 4% for fasting insulin concentration.

To our knowledge, this was the first meta-analysis that directly compared fasting glucose, fasting insulin and HOMA-IR in strength of association with cardiovascular disease.

A number of previous meta-analyses have investigated the association between fasting glucose, fasting insulin or HOMA-IR concentrations and cardiovascular disease by comparing high to low concentrations. Our pooled relative risks of cardiovascular disease (glucose: 1.44, insulin: 1.28, HOMA-IR: 1.44) are within the range of pooled relative risks reported in previous meta-analyses [6], [20]–[22]. Differences in pooled relative risks between meta-analyses may be, for a large part attributed to different cut-off levels of the exposure, leading to different causal contrasts. Further, differences in design aspects of meta-analyses may explain different pooled relative risks. For example, including studies with only fatal events versus studies with fatal and non-fatal events can result in different pooled RR for glucose, since diabetes seems to be a stronger risk factor for fatal than for non-fatal events [23]. Previous studies that investigated sex differences in the association between diabetes and cardiovascular disease found that women with diabetes had a higher relative risk than men with diabetes [3], [24], [25]. The pooled relative risks for an increase of one standard deviation in glucose and insulin were somewhat higher for women than for men, whereas there was less difference in relative risks between sexes for HOMA-IR. It has been proposed that diabetes may induce a more unfavorable cardiovascular risk profile in women than in men and thereby increases cardiovascular disease risk more in women [24], [25]. Another explanation could be that these cardiovascular risk factors are not intermediates, but common causes of both diabetes and cardiovascular disease which may have a stronger effect in women than in men. However, most individual relative risks in this analysis were adjusted for cardiovascular risk factors. Leaving the possibility that there could still be residual confounding, for example by body composition and insulin resistance which are known to differ between men and women [26], [27]. Even if relative risks are truly higher in women than in men, it is important to consider that absolute cardiovascular disease risk are lower [24]. In this meta-analysis, the relative risk of cardiovascular disease was higher for an increase of one standard deviation in HOMA-IR compared to an increase of one standard deviation in glucose or insulin. Animal studies have shown that insulin resistance plays an important role in the early and advanced stages of atherosclerosis, whereas hyperglycemia seems exclusively to be involved in early stages of atherosclerosis [9]. In addition, insulin resistance seems to modify the effect of insulin on the vascular wall; anti-atherogenic in the insulin sensitive state and pro-atherogenic in the insulin resistant state [8]. Unfortunately, it is not clear to what extent these pro-atherogenic mechanism contribute to the development of cardiovascular disease in humans.

A strength of this study is the large number of included studies comprising more than 500,000 participants. Therefore, the pooled effect estimates were not influenced largely by random error and it was possible to investigate different cardiovascular endpoints and sex differences. Secondly, in most studies we were able to calculate the relative risk for an increase of one standard deviation in the exposure. In this way, we adjusted for differences in assays and used cut-off points between studies and could compare the three exposures. Thirdly, we investigate the risk of incident coronary heart disease which is considered to be a homogeneous well-defined cardiovascular disease endpoint [28].

A general limitation of meta-analyses of observational studies is that the result may be a precise, but biased estimate. We assessed the risk of bias per study and performed sensitivity analyses excluding studies with a high risk of bias in a sensitivity analysis. This did not change our results materially. We showed the presence of funnel-plot asymmetry by Egger’s test. Sources of funnel plot asymmetry are publication bias, true heterogeneity of study effects or differences in study quality [17]. Since funnel-plot asymmetry was present for all three exposures, comparing three exposures still seems valid. Most studies included in our meta-analysis measured concentrations only once and are thereby susceptible to random measurement error. Random measurement error of the exposure leads to an attenuation of estimated effects [29]. Moreover, most studies only reported composite cardiovascular disease outcomes which may hamper a causal interpretation of reported risks if the exposure has no uniform effect on the different endpoints [30]. For example, elevated cholesterol concentration is a risk factor for coronary heart disease, but not for stroke [31], [32]. Few studies reported stroke endpoints and associations in women; as a consequence the pooled relative risk of stroke for insulin was based on two studies and the pooled relative risk for HOMA-IR was based on four studies. Finally, we only included studies that measured HOMA-IR, which is a surrogate measure of insulin resistance and mainly reflects hepatic insulin resistance [12]. Therefore, it may not account for the total effect of insulin resistance. However, the application of the gold standard measurement, i.e. the euglycemic hyperinsulinemic clamp which is a measure of peripheral insulin resistance is often not feasible in large epidemiological studies.

More knowledge in the pathofysiology of atherosclerosis should guide type and initiation of treatment. For example, shifting the glucose distribution curve leftwards for the entire population as was postulated previously [33], is only effective when glucose itself is involved in atherosclerosis pathofysiology and when the intervention has a uniform effect in the entire population. However, the addition of HOMA-IR, a marker of insulin resistance to a risk prediction model may improve cardiovascular risk prediction. The addition of a fasting glucose measurement to the Framingham risk score resulted in a slight net reclassification improvement of 1.8% [34]. Whether the addition of HOMA-IR to a risk prediction model, on top of glucose, results in a more accurate reclassification of cardiovascular risk is unknown. Furthermore, this possible benefit should be carefully weighted against the extra costs involved with measuring both glucose and insulin. However, considering the addition of HOMA-IR to a prediction model is important, since many current models aiming to predict cardiovascular events are still not optimal to define high risk groups.

## Supporting Information

Figure S1 **Results of random-effect meta-analyses comparing cardiovascular disease risk in the highest category versus the lowest category.** ^a^One study did not specify sex-specific numbers. I^2^, measure of heterogeneity; 95% CI, 95% confidence interval; CHD, coronary heart disease and is defined as fatal or non-fatal myocardial infarction, or angina pectoris; Stroke is defined as hemorrhagic or ischemic stroke; CVD, cardiovascular disease and is defined as myocardial infarction, angina pectoris, hemorrhagic stroke, ischemic stroke, arrhythmias, congestive heart failure or sudden cardiac death; HOMA-IR, Homeostasis Model Assessment Insulin Resistance.
(TIF)

Table S1 **Search strategy.**
(DOC)

Table S2 **Characteristic of the included studies, organized by exposure.** ^a^All studies are at least adjusted for age and sex. ^b^Mean. ^c^Paris Prospective Study. ^d^Unspecified. ^e^Helsinki Policemen Study. ^f^Analyses stratified by sex; men. ^g^Analyses stratified by sex; women. ^h^Median. ^i^Analyses stratified by the presence of the metabolic syndrome; with the metabolic syndrome. ^j^Analyses stratified by the presence of the metabolic syndrome; without the metabolic syndrome. ^k^Analyses stratified by glomerular filtration rate (GFR); GFR ≥60 ml/min/1.73 m^2^_._^l^Analyses stratified, number represents total for both groups. ^m^Analyses stratified by glomerular filtration rate (GFR); GFR <60 ml/min/1.73 m^2^_._^n^Minimum. ^o^Studies provided additional data stratified by sex. ^p^maximum References are listed in References S1. BP, blood pressure; CVD, (pre-existing) cardiovascular disease; FU, follow-up; HR, hazard ratio; CHD, coronary heart disease; U, unclear; OR, odds ratio; MS, Metabolic Syndrome; GFR, glomerular filtration rate; NGT, normal glucose tolerance; IGT, impaired glucose tolerance; DM, diabetes mellitus.
(DOC)

Table S3 **Risk of bias assessment summarized for three exposures.** HOMA-IR: Homeostasis Model Assessment insulin resistance.
(DOC)

Table S4 **Risk of bias assessment categorized per exposure and per study.** ^a^Paris Prospective Study. ^b^Helsinki Policemen Study. ^c^Percentage includes newly diagnosed diabetes. ^d^Percentage includes known diabetes and newly diagnosed diabetes. References are listed in References S1. DM, diabetes mellitus; CSPE, country specific prevalence estimates; HOMA-IR, Homeostasis Model Assessment Insulin Resistance.
(DOC)

References S1 **References of the included studies.**
(DOC)
