# Supplementary figures and images for: Insulin Resistance and Risk of Incident Cardiovascular Events in Adults without Diabetes: Meta-Analysis
Source: PLoS One. 2012 Dec 28;7(12):e52036. doi: 10.1371/journal.pone.0052036 (PMC3532497; doi:10.1371/journal.pone.0052036)

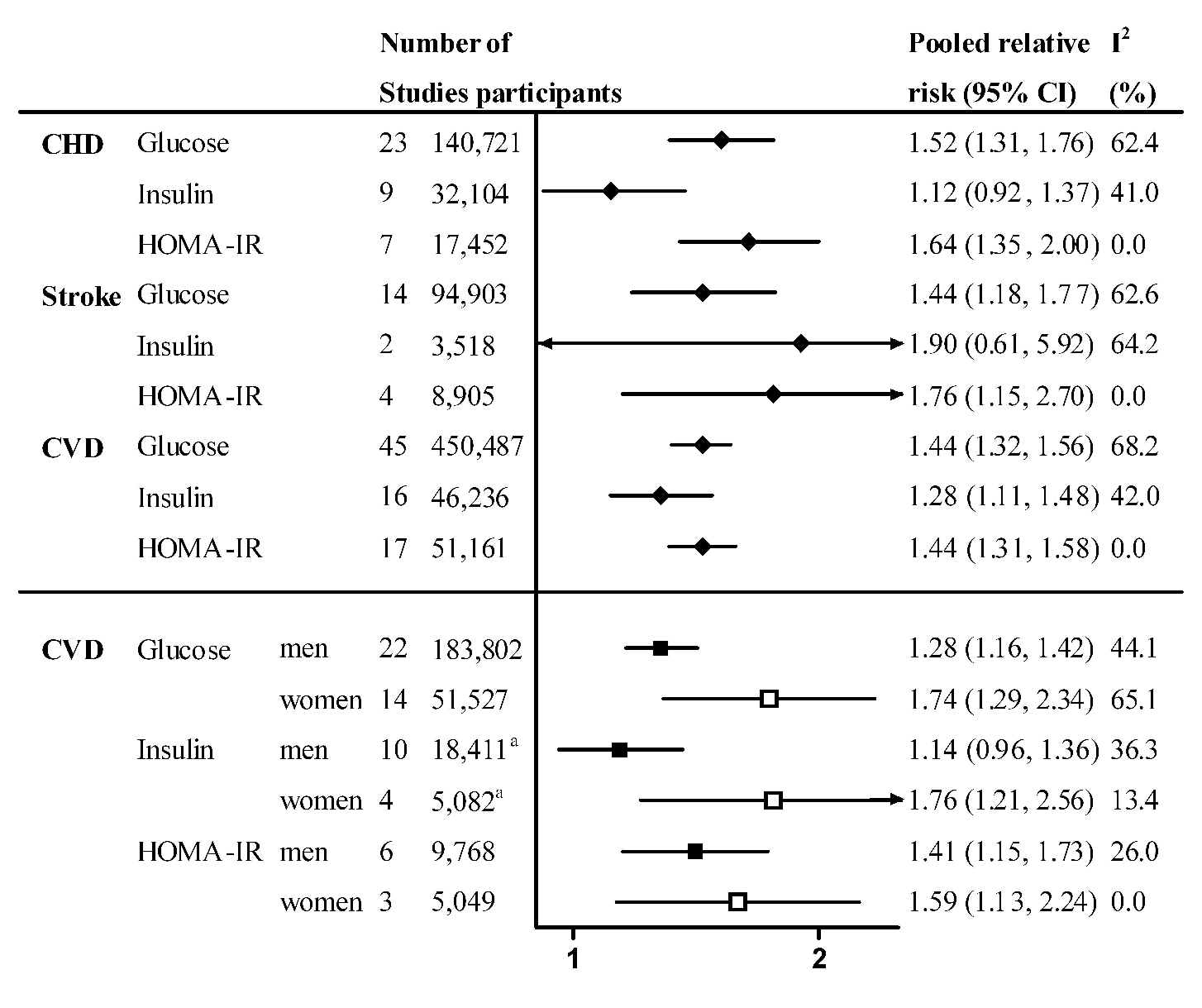

Supplement: Figure S1 — Results of random-effect meta-analyses comparing cardiovascular disease risk in the highest category versus the lowest category. aOne study did not specify sex-specific numbers. I2, measure of heterogeneity; 95% CI, 95% confidence interval; CHD, coronary heart disease and is defined as fatal or non-fatal myocardial infarction, or angina pectoris; Stroke is defined as hemorrhagic or ischemic stroke; CVD, cardiovascular disease and is defined as myocardial infarction, angina pectoris, hemorrhagic stroke, ischemic stroke, arrhythmias, congestive heart failure or sudden cardiac death; HOMA-IR, Homeostasis Model Assessment Insulin Resistance. (TIF) [file pone.0052036.s001.tif]
