# Supplementary material for: Insulin Resistance and Risk of Incident Cardiovascular Events in Adults without Diabetes: Meta-Analysis
Source: PLoS One. 2012 Dec 28;7(12):e52036. doi: 10.1371/journal.pone.0052036 (PMC3532497; doi:10.1371/journal.pone.0052036)
Supplement: Table S1 — Search strategy. (DOC) [file pone.0052036.s002.doc]

| **MEDLINE (OVID-version)** |
| --- |
| (exp Insulin resistance/ OR insulin resistance.ti,ab OR insulin sensitivity.ti,ab OR hyperglycaemia*.ti,ab OR exp hyperglycemia/ OR hyperglycemia*.ti,ab OR exp hyperinsulinism/ OR hyperinsulin*.ti,ab OR glucose intolerance.ti,ab) AND (exp *Cardiovascular Diseases/ OR cardiovascular.ti OR myocardial.ti OR cerebrovascular.ti OR heart disease*.ti OR coronary artery disease*.ti OR stroke.ti OR coronary event*.ti OR cardiovascular mortality.ti OR cardiovascular event*.ti OR vascular disease*.ti) AND ((non-diabetic OR nondiabetic OR non-diabetes OR non-dm OR pre-diabetes OR prediabetes OR pre-diabetic OR prediabetic OR pre-dm OR "subclinical diabetes" OR "subclinical diabetic" OR "sub-clinical diabetes" OR "sub-clinical diabetic" OR normal glucose tolerance OR ngt OR normoglycemia OR normoglycaemia OR normoglycemias OR normoglycaemias OR normoglycemic OR normoglycaemic OR impaired glucose tolerance OR IGT OR "impaired fasting glucose" OR ifg OR without diabet* OR without diagnosed diabet*).mp OR ("free of".ti,ab ADJ10 diabetes.ti,ab) OR (without.mp ADJ5 metabolic syndrome.mp)) AND (exp Adult/ OR adult*.mp) |
| **EMBASE (OVID-version)** |
| (exp *Insulin resistance/ OR insulin resistance.ti OR exp *insulin sensitivity/ OR insulin sensitivity.ti OR hyperglycaem*.ti OR exp *hyperglycemia/ OR hyperglycem*.ti OR exp *hyperinsulinism/ OR hyperinsulin*.ti OR exp *Hyperinsulinemia/ OR glucose intolerance.ti) AND (exp *Cardiovascular Disease/ OR cardiovascular.ti OR myocardial.ti OR cerebrovascular.ti OR heart disease*.ti OR coronary artery disease*.ti OR stroke.ti OR coronary event*.ti OR cardiovascular mortality.ti OR cardiovascular event*.ti OR vascular disease*.ti) AND ((non-diabetic OR nondiabetic OR non-diabetes OR non-dm OR pre-diabetes OR prediabetes OR pre-diabetic OR prediabetic OR pre-dm OR "subclinical diabetes" OR "subclinical diabetic" OR "sub-clinical diabetes" OR "sub-clinical diabetic" OR normal glucose tolerance OR ngt OR normoglycemia OR normoglycaemia OR normoglycemias OR normoglycaemias OR normoglycemic OR normoglycaemic OR impaired glucose tolerance OR IGT OR "impaired fasting glucose" OR ifg OR without diabet* OR without diagnosed diabet*).mp OR impaired glucose tolerance/ OR glucose blood level/) AND (exp Adult/ OR exp Aged/ OR exp Middle aged/ OR adult*.mp) |
| **Web of Science** |
| TI=("Insulin resistance" OR "Insulin sensitivity" OR hyperglycaem* OR hyperglycem* OR hyperinsulin* OR "glucose intoleran*") AND TI=("Cardiovascular disease*" OR "heart disease*" OR "cardiac disease*" OR "myocardial disease*" OR "cerebrovascular disease*" OR "coronary disease*" OR "coronary artery disease*" OR stroke OR "vascular disease*") AND TS=(nondiabetic OR "pre-diabetes" OR "pre-diabetic" OR prediabetes OR prediabetic OR "subclinical diabetes" OR "subclinical diabetic" OR "sub-clinical diabetes" OR "sub-clinical diabetic" OR "normal glucose tolerance" OR ngt OR normoglycemi* OR normoglycaemi* OR "impaired glucose tolerance" OR IGT OR "impaired fasting glucose" OR ifg) |
| **Science Direct**  TITLE(("Insulin resistance" OR "Insulin sensitivity" OR hyperglycaem* OR hyperglycem* OR hyperinsulin* OR "glucose intoleran*") AND ("Cardiovascular disease*" OR "heart disease*" OR "cardiac disease*" OR "myocardial disease*" OR "cerebrovascular disease*" OR "coronary disease*" OR "coronary artery disease*" OR stroke OR "vascular disease*")) AND ("non-diabetic" OR nondiabetic OR "non-diabetes" OR "non-dm" OR "pre-diabetes" OR prediabetes OR "pre-diabetic" OR prediabetic OR "pre-dm" OR "subclinical diabetes" OR "subclinical diabetic" OR "sub-clinical diabetes" OR "sub-clinical diabetic" OR "normal glucose tolerance" OR ngt OR normoglycemi* OR normoglycaemi* OR "impaired glucose tolerance" OR IGT OR "impaired fasting glucose" OR ifg OR (without AND diabet*)) |
| **Cochrane library** |
| ID Search  #1 MeSH descriptor Insulin Resistance explode all trees  #2 MeSH descriptor Hyperglycemia explode all trees  #3 MeSH descriptor Hyperinsulinism explode all trees  #4 (insulin resistance OR insulin sensitivity ):ti  #5 (hyperglycaemi* OR hyperglycemi* OR hyperinsulin* OR glucose intolerance):ti  #6 (hyperglycaemi* OR hyperglycemi*):ti  #7 (hyperinsulin* OR glucose intolerance):ti  #8 (#1 OR #2 OR #3 OR #4 OR #5 OR #6 OR #7)  #9 MeSH descriptor Cardiovascular Diseases explode all trees  #10 (cardiovascular disease OR cardiovascular diseases OR myocardial disease OR myocardial diseases OR cerebrovascular disease OR cerebrovascular diseases OR heart disease OR heart diseases OR coronary artery disease OR coronary artery diseases OR stroke OR coronary event OR coronary events OR cardiovascular mortality OR cardiovascular event OR cardiovascular events OR vascular disease OR vascular diseases):ti  #11 (#8 AND ( #9 OR #10 ))  #12 (non-diabetic OR nondiabetic OR non-diabetes OR non-dm OR pre-diabetes OR prediabetes OR pre-diabetic OR prediabetic OR pre-dm OR "subclinical diabetes" OR "subclinical diabetic" OR "sub-clinical diabetes" OR "sub-clinical diabetic" OR normal glucose tolerance OR ngt OR normoglycemia OR normoglycaemia OR normoglycemias OR normoglycaemias OR normoglycemic OR normoglycaemic OR impaired glucose tolerance OR IGT OR "impaired fasting glucose" OR ifg OR without diabet* OR without diagnosed diabet*)  #13 (#11 AND #12) |
| **PubMed** |
| Strategy 1:  (("Insulin resistance"[mesh] OR "insulin resistance"[tw] OR "insulin sensitivity"[tw] OR hyperglycaemia*[tw] OR "Hyperglycemia"[mesh] OR hyperglycemia*[tw] OR "Hyperinsulinism"[mesh] OR hyperinsulin*[tw] OR "glucose intolerance"[tw]) AND ("Cardiovascular Diseases"[majr] OR cardiovascular[ti] OR myocardial[ti] OR cerebrovascular[ti] OR heart disease*[ti] OR coronary artery disease*[ti] OR stroke[ti] OR coronary event*[ti] OR cardiovascular mortality[ti] OR cardiovascular event*[ti] OR vascular disease*[ti]) AND ("non-diabetic" OR nondiabetic OR "non-diabetes" OR "non-dm" OR "pre-diabetes" OR prediabetes OR "pre-diabetic" OR prediabetic OR "pre-dm" OR "subclinical diabetes" OR "subclinical diabetic" OR "sub-clinical diabetes" OR "sub-clinical diabetic" OR "normal glucose tolerance" OR ngt[tw] OR normoglycemia OR normoglycaemia OR normoglycemias OR normoglycaemias OR normoglycemic OR normoglycaemic OR "impaired glucose tolerance" OR IGT[tw] OR "impaired fasting glucose" OR ifg OR "without diabetes" OR "prediabetic state"[mesh]) AND ("Adult"[mesh] OR adult*))  Strategy 2:  (("Insulin resistance"[mesh] OR "insulin resistance"[tw] OR "insulin sensitivity"[tw] OR hyperglycaemia*[tw] OR "Hyperglycemia"[mesh] OR hyperglycemia*[tw] OR "Hyperinsulinism"[mesh] OR hyperinsulin*[tw] OR "glucose intolerance"[tw]) AND ("Cardiovascular Diseases"[majr] OR cardiovascular[ti] OR myocardial[ti] OR cerebrovascular[ti] OR heart disease*[ti] OR coronary artery disease*[ti] OR stroke[ti] OR coronary event*[ti] OR cardiovascular mortality[ti] OR cardiovascular event*[ti] OR vascular disease*[ti]) AND ("non-diabetic" OR nondiabetic OR "non-diabetes" OR "non-dm" OR "pre-diabetes" OR prediabetes OR "pre-diabetic" OR prediabetic OR "pre-dm" OR "subclinical diabetes" OR "subclinical diabetic" OR "sub-clinical diabetes" OR "sub-clinical diabetic" OR "normal glucose tolerance" OR ngt[tw] OR normoglycemia OR normoglycaemia OR normoglycemias OR normoglycaemias OR normoglycemic OR normoglycaemic OR "impaired glucose tolerance" OR IGT[tw] OR "impaired fasting glucose" OR ifg OR "without diabetes" OR "prediabetic state"[mesh]) NOT ((animal NOT human) OR child OR children)  Strategy 3:  (("Insulin resistance"[mesh] OR "insulin resistance"[tw] OR "insulin sensitivity"[tw] OR hyperglycaemia*[tw] OR "Hyperglycemia"[mesh] OR hyperglycemia*[tw] OR "Hyperinsulinism"[mesh] OR hyperinsulin*[tw] OR "glucose intolerance"[tw] OR "Fasting plasma glucose" OR "Blood Glucose/metabolism"[mesh] OR "elevated glycated hemoglobin" OR "HbA(1c)" OR "Blood Glucose"[majr] OR "Blood Glucose/analysis"[mesh] OR "fasting glucose" OR "Hypoglycemic Agents/therapeutic use"[mesh] OR "Insulin/therapeutic use"[mesh] OR "glycated hemoglobin" OR "Hemoglobin A, Glycosylated/metabolism"[majr] OR "Insulin/blood"[majr] OR "Glucose/metabolism"[majr] OR glucometabolic[tw] OR "Blood Glucose"[mesh:noexp] OR "blood glucose"[tiab]) AND ("Cardiovascular Diseases"[majr] OR cardiovascular[ti] OR myocardial[ti] OR cerebrovascular[ti] OR heart disease*[ti] OR coronary artery disease*[ti] OR stroke[ti] OR coronary event*[ti] OR cardiovascular mortality[ti] OR cardiovascular event*[ti] OR vascular disease*[ti] OR ("Coronary Disease"[mesh] AND (non-diabetic*[ti] OR nondiabetic*[ti])) OR "Coronary Disease/epidemiology"[mesh]) AND ("non-diabetic" OR nondiabetic OR "non-diabetes" OR "non-dm" OR "pre-diabetes" OR prediabetes OR "pre-diabetic" OR prediabetic OR "pre-dm" OR "subclinical diabetes" OR "sub-clinical diabetes" OR "normal glucose tolerance" OR ngt[tw] OR normoglycemia OR normoglycaemia OR normoglycaemias OR normoglycemic OR normoglycaemic OR "impaired glucose tolerance" OR IGT[tw] OR "impaired fasting glucose" OR ifg OR "without diabetes" OR "prediabetic state"[mesh] OR (("Reference Values"[mesh] OR "Population Surveillance"[mesh] OR "Socioeconomic Factors"[mesh] OR "Disease-Free Survival"[mesh]) AND ("Heart Disease"[ti] OR "heart diseases"[ti] OR coronary[ti] OR chd[ti] OR "myocardial infarction"[ti] OR cardiovascular[ti])) OR nondiabetics OR "Comorbidity"[mesh] OR "serum insulin level" OR "serum insulin levels" OR "glucose tolerance"[ti] OR "non-diabetics" OR "initial glucose level" OR "initial glucose levels" OR "population studies"[ti] OR "population study"[ti] OR "Body weight"[ti] OR predict[ti] OR predictor[ti] OR "non-insulin-resistant subjects" OR "all-causes"[ti] OR "excluding diabetes" OR (("control subjects" OR controls[tw]) AND ("metabolic syndrome"[ti] OR diabetes[ti] OR insulin[ti]) AND (cardiovascular[ti] OR coronary[ti] OR cardiac[ti] OR myocardial[ti])) OR "Population"[majr] OR "general population" OR "2-hour glucose" OR (diabetes[ti] AND ("risk marker"[ti] OR "risk markers"[ti])) OR "population survey" OR "community-based sample" OR (Known[tiab] diabetics[tiab] excluded[tiab]) OR (excluding[tiab] men[tiab] diabetes[tiab]) OR "whole population sample") AND ("Adult"[mesh] OR adult*)) |
