# Supplementary material for: Insulin Resistance and Risk of Incident Cardiovascular Events in Adults without Diabetes: Meta-Analysis
Source: PLoS One. 2012 Dec 28;7(12):e52036. doi: 10.1371/journal.pone.0052036 (PMC3532497; doi:10.1371/journal.pone.0052036)
Supplement: Table S2 — Characteristic of the included studies, organized by exposure. aAll studies are at least adjusted for age and sex. bMean. cParis Prospective Study. dUnspecified. eHelsinki Policemen Study. fAnalyses stratified by sex; men. gAnalyses stratified by sex; women. hMedian. iAnalyses stratified by the presence of the metabolic syndrome; with the metabolic syndrome. jAnalyses stratified by the presence of the metabolic syndrome; without the metabolic syndrome. kAnalyses stratified by glomerular filtration rate (GFR); GFR ≥60 ml/min/1.73 m2 . lAnalyses stratified, number represents total for both groups. mAnalyses stratified by glomerular filtration rate (GFR); GFR <60 ml/min/1.73 m2 . nMinimum. oStudies provided additional data stratified by sex. pmaximum References are listed in References S1. BP, blood pressure; CVD, (pre-existing) cardiovascular disease; FU, follow-up; HR, hazard ratio; CHD, coronary heart disease; U, unclear; OR, odds ratio; MS, Metabolic Syndrome; GFR, glomerular filtration rate; NGT, normal glucose tolerance; IGT, impaired glucose tolerance; DM, diabetes mellitus. (DOC) [file pone.0052036.s003.doc]

| **Source** | **Study design** | **Number** | **Men (%)** | **Study population** | **Age (range)** | **Categories** | **Effect measure** | **Adjustments^a^** | | | | | | **End point** | **Events**  **(n)** | **FU (yr) (mean)** |
| --- | --- | --- | --- | --- | --- | --- | --- | --- | --- | --- | --- | --- | --- | --- | --- | --- |
|  |  |  |  |  |  |  |  | **Smoking** | **BP** | **Lipids** | **Adiposity** | **CVD** | **Other** |  |  |  |
| **Glucose** |  |  |  |  |  |  |  |  |  |  |  |  |  |  |  |  |
| Baba, 2007 [35] | Cohort | 2,024 | 38 | Atomic bomb survivors | 62.7^b^ | Cut-off | HR | Yes | No | No | No | No | Yes | Fatal & non-fatal CHD | 49 | U |
| Balkau, 1998^c^ [36] | Cohort | 6,629 | 100 | Civil servants | 44-55 | 40-iles | HR | Yes | Yes | Yes | Yes | Yes | Yes | Fatal CVD  Fatal CHD  Fatal Stroke | 446  284  76 | 20^d^ |
| Balkau, 1998^e^ [36] | Cohort | 631 | 100 | Policemen | 44-55 | 40-iles | HR | Yes | Yes | Yes | Yes | Yes | Yes | Fatal CVD  Fatal CHD  Fatal Stroke | 99  61  17 | 20^d^ |
| Barrett-Connor, 1984^f^ [37] | Cohort | 1,610 | 100 | Upper-middle class individuals | 40-79 | Cut-off | HR | Yes | Yes | Yes | Yes | No | No | Fatal CVD  Fatal CHD | 166  114 | 9^d^ |
| Barrett-Connor, 1984^g^ [37] | Cohort | 2,015 | 0 | Upper-middle class individuals | 40-79 | Cut-off | HR | Yes | Yes | Yes | Yes | No | No | Fatal CVD  Fatal CHD | 69  36 | 9^d^ |
| Bjornholt, 1999 [38] | Cohort | 1,973 | 100 | Employees | 40-59 | Quartiles | HR | U | U | U | U | U | U | Fatal CVD | 242 | 22^d^ |
| Brunner, 2010 [39] | Cohort | 6,868 | 67 | Servants | 40-55 | Cut-off | HR | Yes | Yes | Yes | No | No | Yes | Fatal & non-fatal CHD | 443 | 11.3 |
| Cederberg, 2010^f^ [40] | Cohort | 223 | 100 | General population | 60-64 | Cut-off | OR | No | No | No | No | No | No | Fatal & non-fatal CVD | 95 | 9.7 |
| Cederberg, 2010^g^ [40] | Cohort | 330 | 0 | General population | 60-64 | Quintiles | OR | No | No | No | No | No | No | Fatal & non-fatal CVD | 113 | 9.7 |
| Chien, 2009 [41] | Cohort | 16,590 | 60 | Attendants health examination | 51.9^b^ | Quintiles | HR | Yes | Yes | Yes | Yes | No | Yes | Fatal CVD | 95 | 3.5^h^ |
| Dekker, 2005^f^ [42] | Cohort | 615 | 100 | General population | 50-75 | Quartiles | HR | No | No | No | No | No | No | Fatal & non-fatal CVD | 132 | 10^d^ |
| Dekker, 2005^g^ [42] | Cohort | 749 | 0 | General population | 50-75 | Quartiles | HR | No | No | No | No | No | No | Fatal & non-fatal CVD | 95 | 10^d^ |
| Doi, 2010^f^ [43] | Cohort | 1,037 | 100 | General population | 40-79 | Cut-off | HR | Yes | Yes | Yes | Yes | Yes | Yes | Fatal & non-fatal CHD  Fatal & non-fatal Stroke | 75  61 | 14^d^ |
| Doi, 2010^g^ [43] | Cohort | 1,384 | 0 | General population | 40-79 | Cut-off | HR | Yes | Yes | Yes | Yes | Yes | Yes | Fatal & non-fatal CHD  Fatal & non-fatal Stroke | 37  71 | 14^d^ |
| Eberly, 2006^i^ [44] | Cohort | 4,625 | 100 | Trial participants with the MS | 35-57 | Cut-off | HR | Yes | Yes | Yes | Yes | Yes | Yes | Fatal CVD | 899 | 18.4^h^ |
| Eberly, 2006^j^ [44] | Cohort | 4,611 | 100 | Trial participants without the MS | 35-57 | Cut-off | HR | Yes | Yes | Yes | Yes | Yes | Yes | Fatal CVD | 846 | 18.4^h^ |
| Ford, 2004 [45] | Cohort | 2,431 | 46 | General population | 30-75 | Cut-off | HR | Yes | Yes | Yes | Yes | Yes | Yes | Fatal CVD  Fatal CHD  Fatal Stroke | 200  147  67 | 13.5 |
| Girman, 2004 [46] | Cohort | 3,188 | 85 | Trial participants | 58^b^ | Cut-off | HR | No | No | No | No | No | No | Fatal & non-fatal CVD  Fatal & non-fatal CHD | U  U | 5^d^ |
| Hailpern, 2006^k^ [47] | Cohort | 9,918^l^ | 62 | Persons with untreated hypertension, GFR ≥ 60 ml/min/1.73 m^2^ | ≥18 | Cut-off | HR | Yes | Yes | Yes | Yes | Yes | Yes | Fatal CVD  Fatal CHD  Fatal Stroke | 503^l^  337^l^  61^l^ | 9.6 |
| Hailpern, 2006^m^ [47] | Cohort |  | 62 | Persons with untreated hypertension, GFR < 60 ml/min/1.73 m^2^ | ≥18 | Cut-off | HR | Yes | Yes | Yes | Yes | Yes | Yes | Fatal CVD  Fatal CHD  Fatal Stroke | 503^l^  337^l^  61^l^ | 9.6 |
| Henry, 2002 [48] | Cohort | 63,443 | 100 | Attendants health examination | 21-60 | Cut-off | HR | Yes | Yes | Yes | Yes | Yes | No | Fatal CVD | 171 | 8^m^ |
| Ho, 2008 [49] | Cohort | 30,365 | 100 | Attendants health examination | 44^b^ | Cut-off | HR | No | Yes | Yes | Yes | No | No | Fatal CVD | 527 | 13.6^h^ |
| Hsu, 2007 [50] | Cohort | 4,888 | 100 | General population | ≥30 | Cut-off | HR | Yes | No | No | No | No | No | Fatal CVD | 246^l^ | 10.6 |
| Hsu, 2007 [50] | Cohort | 6,170 | 0 | General population | ≥30 | Cut-off | HR | Yes | No | No | No | No | No | Fatal CVD | 246^l^ | 10.6 |
| Hunt, 2004 [51] | Cohort | 2,617 | 44 | General population | 25-64 | Cut-off | HR | No | No | No | No | No | Yes | Fatal CVD | 84 | 12.7 |
| Hwang, 2009^f^ [52] | Cohort | 1,761 | 100 | Volunteers health care program | 20-78 | Cut-off | OR | Yes | No | Yes | No | No | No | Fatal & non-fatal CVD  Fatal & non-fatal CHD  Fatal & non-fatal Stroke | 106  39  70 | 8.7 |
| Hwang, 2009^g^ [52] | Cohort | 674 | 0 | Volunteers health care program | 20-78 | Cut-off | OR | Yes | No | Yes | No | No | No | Fatal & non-fatal CVD  Fatal & non-fatal CHD  Fatal & non-fatal Stroke | 30  8  23 | 8.7 |
| Jeppesen, 2007 [53] | Cohort | 2,493 | 49 | General population | 41-71 | Cut-off | HR | Yes | No | Yes | No | No | No | Fatal & non-fatal CVD | 233 | 9.4^h^ |
| Juutilainen, 2006^f^ [54] | Cohort | 574 | 100 | General population | 45-64 | Cut-off | HR | Yes | No | Yes | No | No | Yes | Fatal CVD | 75 | 18^d^ |
| Juutilainen, 2006^g^ [54] | Cohort | 707 | 0 | General population | 45-64 | Cut-off | HR | Yes | No | Yes | No | No | Yes | Fatal CVD | 23 | 18^d^ |
| Khang, 2010^o^ [55] | Cohort | 9,791 | 45 | General population | ≥20 | Cut-off | HR | No | Yes | Yes | Yes | No | Yes | Fatal & non-fatal CVD  Fatal & non-fatal CHD  Fatal & non-fatal Stroke | 288  122  184 | 5.8 |
| Kokubo, 2010^o^ [56] | Cohort | 5,321 | 47 | General population | 30-79 | Cut-off | HR | Yes | Yes | Yes | Yes | No | Yes | Fatal & non-fatal CVD  Fatal & non-fatal CHD  Fatal & non-fatal Stroke | 364  166  198 | 11.7^h^ |
| Lapidus, 1985 [57] | Cohort | 1,424 | 0 | General population | 38-60 | Cut-off | OR | No | No | No | No | No | No | Fatal & non-fatal CHD  Fatal & non-fatal Stroke | 23  13 | 12^d^ |
| Liu, 2007 [58] | Cohort | 30,378 | 53 | General population | 35-64 | Cut-off | HR | Yes | No | Yes | No | No | Yes | Fatal & non-fatal CVD  Fatal & non-fatal CHD  Fatal & non-fatal Stroke | 738  U  U | 10^d^ |
| Marin, 2006^o^ [59] | Cohort | 4,434 | 62 | Patients from primary care centers | 25-99 | Cut-off | HR | Yes | Yes | Yes | Yes | No | Yes | Fatal & non-fatal CHD | 147 | 5.0 |
| Nakanishi, 2004 [60] | Cohort | 6,182 | 100 | Employees government | 35-39 | Cut-off | HR | Yes | No | No | No | No | Yes | Non-fatal CVD | 70 | 7^d^ |
| Nichols, 2009 [61] | Cohort | 20,226 | 48 | Health insurance members with NGT or IGT | 64 | Cut-off | HR | Yes | Yes | Yes | Yes | Yes | Yes | (Fatal?) & non-fatal CVD | U | 6.6 |
| Nilsson, 2007 [62] | Cohort | 5,047 | 40 | General population | 46-68 | Cut-off | HR | No | No | No | No | No | No | Fatal & non-fatal CVD | 176 | 10.7 |
| Preiss, 2010 [63] | Cohort | 6,447 | 100 | Trial participants | 45-64 | Quintiles | HR | Yes | No | Yes | Yes | Yes | Yes | Fatal & non-fatal CVD  Fatal & non-fatal CHD  Fatal & non-fatal Stroke | 2,381  1,474  405 | 15^d^ |
| Sarwar, 2010 [64] | Cohort | 18,333 | 49 | General population | 32-61 | Cut-off | HR | Yes | Yes | Yes | Yes | No | Yes | Fatal & non-fatal CHD | 4,490 | 23.5 |
| Sattar, 2008 [65] | Cohort | 3,361 | 47 | Trial participants | 70-82 | Cut-off | HR | No | No | No | No | No | Yes | Fatal & non-fatal CVD | 434 | 3.2 |
| Schillaci, 2004 [66] | Cohort | 1,742 | 55 | Patients with hypertension | 50.3 | Cut-off | HR | Yes | Yes | Yes | No | No | Yes | Fatal & non-fatal CVD | 162 | 4.1 |
| Selvin, 2010 [67] | Cohort | 11,092 | 42 | General population | 47-69 | Cut-off | HR | Yes | Yes | Yes | Yes | No | Yes | Fatal & non-fatal CHD  Fatal & non-fatal Stroke | 1,198  358 | 14^h^ |
| Shin, 2009^o^ [68] | Cohort | 57,237 | 57 | Attendants health examination | 40-89 | Cut-off | HR | Yes | Yes | Yes | No | No | Yes | Fatal CVD | 129 | 5.6 |
| Simons, 2000^f^ [69] | Cohort | 1,045 | 100 | General population | 60-79 | Quartiles | HR | U | U | U | U | U | U | Fatal & non-fatal CHD  Fatal & non-fatal Stroke | 339  139 | 9.4^h^ |
| Simons, 2000^g^ [69] | Cohort | 1,374 | 0 | General population | 60-79 | Quartiles | HR | U | U | U | U | U | U | Fatal & non-fatal CHD  Fatal & non-fatal Stroke | 339  141 | 9.4^h^ |
| Smith, 2002 [70] | Cohort | 4,014 | 40 | General population | 45-64 | Quintiles | HR | Yes | Yes | Yes | Yes | No | Yes | Fatal & non-fatal CVD | 764 | 8.5^h^ |
| Tai, 2004 [71] | Cohort | 5,091 | 50 | General population and volunteers following health intervention program | U | Cut-off | HR | No | No | No | No | No | Yes | Fatal & non-fatal CHD | 128 | 8^d^ |
| Thomas, 2007^o^ [72] | Cohort | 2,863 | 50 | General population | 25-74 | Cut-off | HR | Yes | No | No | No | No | Yes | Fatal CVD | 30 | 8.5 |
| Tsai, 2008 [73] | Cohort | 35,259 | 66 | Attendants health examination | ≥40 | Cut-off | HR | No | No | No | No | No | No | Fatal CVD | 468 | 15^h^ |
| Wang, 2007 [74] | Cohort | 541 | 50 | Participants with a high risk for DM | ≥25 | Cut-off | OR | Yes | No | Yes | No | No | Yes | Non-fatal CHD | 236 | 5^d^ |
| Watanabe, 2008 [75] | Cohort | 28,449 | 34 | Attendants health examination | ≥20 | Cut-off | HR | No | No | No | No | No | No | Non-fatal CVD | 265 | 4.5 |
| Wilson, 2005 [76] | Cohort | 3,323 | 47 | Offspring Framingham Study | 22-81 | Cut-off | HR | No | No | No | No | No | No | Fatal & non-fatal CVD  Fatal & non-fatal CHD | 174  107 | 8^p^ |
| Yarnell, 1998 [77] | Cohort | 4,197 | 100 | General population | 45-63 | Quintiles | OR | Yes | Yes | Yes | No | Yes | Yes | Fatal & non-fatal CHD | 492 | 9.7^d^ |
| Zhang, 2009 [78] | Cohort | 2,173 | 45 | General population | ≥45 | Cut-off | HR | Yes | No | No | No | No | Yes | Fatal & non-fatal Stroke | 52 | 4.6^h^ |
| **Insulin** |  |  |  |  |  |  |  |  |  |  |  |  |  |  |  |  |
| Bonara, 2007 [79] | Cohort | 839 | 50 | General population | 40-79 | Quartiles | HR | Yes | Yes | Yes | Yes | No | Yes | Fatal & non-fatal CVD | 118 | 15^d^ |
| Chien, 2008 [80] | Cohort | 2,165 | 44 | General population | ≥35 | Quartiles | HR | Yes | No | No | Yes | No | Yes | Fatal & non-fatal CHD | 166 | 10.5^h^ |
| Dekker, 2005^f^ [42] | Cohort | 615 | 100 | General population | 50-75 | Quartiles | HR | No | No | No | No | No | No | Fatal & non-fatal CVD | 132 | 10^d^ |
| Dekker, 2005^g^ [42] | Cohort | 749 | 0 | General population | 50-75 | Quartiles | HR | No | No | No | No | No | No | Fatal & non-fatal CVD | 95 | 10^d^ |
| Folsom, 1997^f^ [81] | Cohort | 13,446^l^ | 100 | General population | 45-64 | Cut-off | HR | Yes | Yes | Yes | Yes | No | Yes | Fatal & non-fatal CHD | 209 | U |
| Folsom, 1997^g^ [81] | Cohort | 13,446^l^ | 0 | General population | 45-64 | Cut-off | HR | Yes | Yes | Yes | Yes | No | Yes | Fatal & non-fatal CHD | 96 | U |
| Jeppesen, 2010 [82] | Cohort | 2,265 | 49 | General population | 41-71 | Quartiles | HR | Yes | Yes | Yes | No | No | No | Fatal CVD  Fatal & non-fatal CHD | 119  169 | 12.6^h^ |
| Juutilianen, 2006^f^ [54] | Cohort | 574 | 100 | General population | 45-64 | Quartiles | HR | Yes | No | Yes | No | No | Yes | Fatal CVD | 75 | 18^d^ |
| Juutilianen, 2006^g^[54] | Cohort | 707 | 0 | General population | 45-64 | Quartiles | HR | Yes | No | Yes | No | No | Yes | Fatal CVD | 23 | 18^d^ |
| Liu, 1992 [83] | Cohort | 589 | 35 | General population | ≥25 | Deciles | HR | No | No | No | No | No | No | Non-fatal CHD | 16 | 6.7 |
| Nakamura, 2010 [84] | Cohort | 2,548 | 100 | General population | 35-59 | Quartiles | HR | Yes | Yes | Yes | No | No | Yes | Fatal & non-fatal CVD  Fatal & non-fatal CHD  Fatal & non-fatal Stroke | 58  33  25 | 11 |
| Nilsson, 2003 [85] | Cohort | 6,074 | 100 | General population | 25-64 | Deciles | HR | Yes | Yes | Yes | Yes | No | Yes | Fatal & non-fatal CHD | 677 | 19 |
| Orchard, 1994 [86] | Nested  case-control | 622 | 100 | Men with high risk of CVD | 35-57 | Quartiles | OR | Yes | Yes | Yes | No | No | Yes | Fatal & non-fatal CHD | 208 | U |
| Oterdoom, 2009^f^ [87] | Cohort | 3,290 | 100 | General population | 28-75 | Quartiles | HR | No | No | No | No | No | No | Fatal & non-fatal CVD | 242 | 7.5^d^ |
| Oterdoom, 2009^g^ [87] | Cohort | 3,626 | 0 | General population | 28-75 | Quartiles | HR | No | No | No | No | No | No | Fatal & non-fatal CVD | 98 | 7.5^d^ |
| Pyorala, 1998 [88] | Cohort | 970 | 100 | Policemen | 34-64 | Quintiles | HR | Yes | Yes | No | Yes | No | No | Fatal & non-fatal stroke | 70 | 22.3^h^ |
| Rutter, 2005 [89] | Cohort | 2,898 | 45 | Offspring Framingham study | 26-82 | Quartiles | HR | Yes | No | Yes | Yes | No | Yes | Fatal & non-fatal CVD | 186 | 6.7^h^ |
| St-Pierre, 2005 [90] | Cohort | 1,824 | 100 | General population | 34-64 | Quartiles | HR | Yes | No | No | No | No | Yes | Fatal & non-fatal CHD | 284 | 13^d^ |
| Wang, 2007 [74] | Cohort | 541 | 50 | Participants with a high risk of DM | ≥25 | Quartiles | OR | Yes | No | Yes | No | No | Yes | Non-fatal CHD | 236 | 5^d^ |
| Yarnell, 1998 [77] | Cohort | 1,896 | 100 | General population | 45-63 | Quintiles | OR | Yes | Yes | Yes | No | Yes | Yes | Fatal & non-fatal CHD | 221 | 9.7^d^ |
| **HOMA-IR** |  |  |  |  |  |  |  |  |  |  |  |  |  |  |  |  |
| Arnlov, 2010 [91] | Cohort | 958 | 100 | General population | 50 | Quartiles | HR | Yes | No | Yes | No | No | No | Fatal & non-fatal CVD | 318 | 30^h^ |
| Barr, 2010 [92] | Cohort | 6,942 | 55 | General population | ≥25 | Quintiles | HR | Yes | Yes | Yes | Yes | Yes | Yes | Fatal & non-fatal CVD | 225 | 5^h^ |
| Bonora, 2007 [79] | Cohort | 839 | 50 | General population | 40-79 | Quartiles | HR | Yes | Yes | Yes | Yes | No | Yes | Fatal & non-fatal CVD | 118 | 15^d^ |
| Chien, 2008 [80] | Cohort | 2,165 | 44 | General population | ≥35 | Quartiles | HR | Yes | No | No | Yes | No | Yes | Fatal & non-fatal CHD | 166 | 10.5^h^ |
| Dekker, 2005^f^ [42] | Cohort | 615 | 100 | General population | 50-75 | Quartiles | HR | No | No | No | No | No | No | Fatal & non-fatal CVD | 132 | 10^d^ |
| Dekker, 2005^g^ [42] | Cohort | 749 | 0 | General population | 50-75 | Quartiles | HR | No | No | No | No | No | No | Fatal & non-fatal CVD | 95 | 10^d^ |
| Hanley, 2002 [93] | Cohort | 2,413 | 43 | General population | 25-64 | Quintiles | OR | Yes | Yes | Yes | Yes | No | Yes | Fatal & non-fatal CVD  Fatal & non-fatal CHD  Fatal & non-fatal Stroke | 187  U  U | 7.5^h^ |
| Hedblad, 2002 [94] | Cohort | 4,748 | 39 | General population | 46-68 | Quartiles | HR | Yes | Yes | Yes | Yes | No | Yes | Fatal & non-fatal CHD | 62 | 5^h^ |
| Hwang, 2009^f^ [52] | Cohort | 1,761 | 100 | Volunteers health care program | 20-78 | Tertiles | OR | Yes | No | Yes | No | No | No | Fatal & non-fatal CVD  Fatal & non-fatal CHD  Fatal & non-fatal Stroke | 106  39  70 | 8.7 |
| Hwang, 2009^g^ [52] | Cohort | 674 | 0 | Volunteers health care program | 20-78 | Tertiles | OR | Yes | No | Yes | No | No | No | Fatal & non-fatal CVD  Fatal & non-fatal CHD  Fatal & non-fatal Stroke | 30  8  23 | 8.7 |
| Isomaa, 2001 [95] | Cohort | 4,483 | 48 | Families | 35-70 | Quartiles | OR | Yes | No | Yes | No | No | No | Fatal CVD | 209 | 6.9^h^ |
| Jeppesen, 2010 [82] | Cohort | 2,265 | 49 | General population | 41-71 | Quartiles | HR | Yes | Yes | Yes | No | No | No | Fatal CVD  Fatal & non-fatal CHD | 119  169 | 12.6^h^ |
| Nakamura, 2010 [84] | Cohort | 2,548 | 100 | Factory workers | 35-59 | Quartiles | HR | Yes | Yes | Yes | No | No | Yes | Fatal & non-fatal CVD  Fatal & non-fatal CHD  Fatal & non-fatal Stroke | 58  33  25 | 11 |
| Nilsson, 2007 [62] | Cohort | 5,047 | 40 | General population | 46-68 | Quartiles | HR | No | No | No | No | No | No | Fatal & non-fatal CVD | 176 | 10.7 |
| Onat, 2006 [96] | Cohort | 1,348 | 44 | General population | 52.2^b^ | Quartiles | OR | No | No | Yes | Yes | No | Yes | Fatal & non-fatal CHD | 147 | 2.2 |
| Oterdoom, 2009^f^ [87] | Cohort | 3,290 | 100 | General population | 28-75 | Quartiles | HR | No | No | No | No | No | No | Fatal & non-fatal CVD | 242 | 7.5^d^ |
| Oterdoom, 2009^g^ [87] | Cohort | 3,626 | 0 | General population | 28-75 | Quartiles | HR | No | No | No | No | No | No | Fatal & non-fatal CVD | 98 | 7.5^d^ |
| Resnick, 2003 [97] | Cohort | 2,283 | 43 | American Indians from 12 tribes | 45-74 | Tertiles | HR | Yes | Yes | Yes | Yes | No | Yes | Fatal & non-fatal CVD | 181 | 7.6 |
| Rundek, 2010 [98] | Cohort | 1,509 | 36 | General population | ≥39 | Quartiles | HR | Yes | Yes | Yes | Yes | No | Yes | Fatal & non-fatal CVD  Fatal & non-fatal CHD  Fatal & non-fatal Stroke | 180  45  46 | 8.5 |
| Rutter, 2005 [89] | Cohort | 2,898 | 45 | Offspring Framingham study | 22-81 | Quartiles | HR | Yes | No | Yes | No | No | No | Fatal & non-fatal CVD | 186 | 6.7^h^ |
