# Supplementary material for: Insulin Resistance and Risk of Incident Cardiovascular Events in Adults without Diabetes: Meta-Analysis
Source: PLoS One. 2012 Dec 28;7(12):e52036. doi: 10.1371/journal.pone.0052036 (PMC3532497; doi:10.1371/journal.pone.0052036)
Supplement: Table S3 — Risk of bias assessment summarized for three exposures. HOMA-IR: Homeostasis Model Assessment insulin resistance. (DOC) [file pone.0052036.s004.doc]

|  | **Exposure** |  |  |
| --- | --- | --- | --- |
| **Potential sources of bias** | **Glucose**  (45 studies) | **Insulin**  (16 studies) | **HOMA-IR**  (17 studies) |
| Oversampling of overt diabetes at baseline  No  Yes  Unclear | 87%  0%  13% | 100%  0%  0% | 94%  0%  6% |
| Presence of outcome at baseline  No  Yes  Unclear | 71%  2%  27% | 75%  0%  25% | 44%  0%  56% |
| Unspecified definition of fasting  No  Yes | 62%  38% | 75%  25% | 82%  18% |
| More than 10% missing data for the exposure  No  Yes  Unclear | 25%  11%  64% | 56%  25%  19% | 47%  18%  35% |
| Unreliable outcome assessment  No  Yes  Unclear | 73%  16%  11% | 50%  31%  19% | 65%  24%  11% |
| More than 10% loss to follow-up  No  Yes  Unclear | 31%  9%  60% | 31%  13%  56% | 17%  24%  59% |
