# Supplementary material for: Insulin Resistance and Risk of Incident Cardiovascular Events in Adults without Diabetes: Meta-Analysis
Source: PLoS One. 2012 Dec 28;7(12):e52036. doi: 10.1371/journal.pone.0052036 (PMC3532497; doi:10.1371/journal.pone.0052036)
Supplement: Table S4 — Risk of bias assessment categorized per exposure and per study. aParis Prospective Study. bHelsinki Policemen Study. cPercentage includes newly diagnosed diabetes. dPercentage includes known diabetes and newly diagnosed diabetes. References are listed in References S1. DM, diabetes mellitus; CSPE, country specific prevalence estimates; HOMA-IR, Homeostasis Model Assessment Insulin Resistance. (DOC) [file pone.0052036.s005.doc]

| **Source** | **Overt DM at baseline (%)**  **(CSPE)** | **Presence of outcome at baseline (%)** | **Unspecified definition of fasting** | **Missing data for the exposure (%)** | **Endpoint assessment** | **Loss to follow-up (%)** |
| --- | --- | --- | --- | --- | --- | --- |
| **Glucose** |  |  |  |  |  |  |
| Baba, 2007 [35] | Unclear (11%) | 0% | Unclear | Unclear | Self-reports, electrocardiograms, medical records, death certificates | Unclear |
| Balkau ,1998^a^ [36] | 0% (7%) | 0% | Overnight | Unclear | Administrative departments employer, relatives, medical records | 5% |
| Balkau, 1998^b^ [36] | 0% (9%) | 0% | 12 hours | Unclear | Death certificates, autopsy reports, medical records | 0% |
| Barrett-Connor, 1984 [37] | 0%^c^ (11%) | 0% | 12 hours | 6% | Death certificates | 0% |
| Bjornholt, 1999 [38] | 0%^d^ (6%) | 0% | 12 hours | Unclear | Death certificates | Unclear |
| Brunner, 2010 [39] | 0% (7%) | 0% | Unclear | 5% | Medical records, death certificates | Unclear |
| Cederberg, 2010 [40] | 0% (9%) | Unclear | 12 hours | Unclear | Hospital discharge diagnoses, death certificates | Unclear |
| Chien, 2009 [41] | 0%^d^ (10%) | 0% | Unclear | Unclear | Death certificates | Unclear |
| Dekker, 2005 [42] | 0%^c^ (7%) | Unclear | Overnight | 3% | Medical records, fatal events: unclear | 25% |
| Doi, 2010 [43] | ♂:15%^c^ (11%)  ♀:10%^c^ | 0% | Overnight | 4% | Clinical examinations, autopsies | 0% |
| Eberly, 2006 [44] | Unclear (11%) | 0% | 12 hours | 1% | Death certificates | Unclear |
| Ford, 2004 [45] | 3% (11%) | 0% | 10 hours | 74% | Death certificates | 2% |
| Girman, 2004 [46] | 0%^c^ (11%) | Unclear | Unclear | Unclear | Unclear | Unclear |
| Hailpern, 2006 [47] | 0%^c^ (11%) | 0% | Unclear | Unclear | Death certificates | Unclear |
| Henry, 2002 [48] | 0% (7%) | 0% | Unclear | Unclear | Death certificates | Unclear |
| Ho, 2008 [49] | 5% (11%) | 0% | Unclear | Unclear | Death certificates | Unclear |
| Hsu, 2008 [50] | 8%^d^ (10%) | 0% | Overnight | Unclear | Death certificates | Unclear |
| Hunt, 2004 [51] | 0%^c^ (11%) | 0% | 12 hours | 4% | Death certificates | 1% |
| Hwang, 2009 [52] | 7%^c^ (9%) | Unclear | Overnight | 30% | Self-reports and medical records, fatal events: unclear | Unclear |
| Jeppesen, 2007 [53] | 3% (8%) | 0% | Overnight | Unclear | Register of hospitalizations, death certificates | Unclear |
| Juutilainen, 2006 [54] | 0% (9%) | 0% | 12 hours | Unclear | Death certificates | Unclear |
| Khang, 2010 [55] | 0% (9%) | 0% | Unclear | 13% | Hospital admissions and discharges, death certificates | Unclear |
| Kokubo, 2010 [56] | Unclear (11%) | Unclear | Unclear | 3% | Medical records, death certificates | 10% |
| Lapidus, 1985 [57] | 1% (6%) | 0% | Overnight | Unclear | Medical records, death certificates | 3% |
| Liu, 2007 [58] | 7%^c^ (9%) | Unclear | Unclear | Unclear | WHO MONICA protocol | 14% |
| Marin, 2006 [59] | 11%^c^ (8%) | 0% | 10 hours | Unclear | Medical records, death certificates | 2% |
| Nakanishi, 2004 [60] | 0%^d^ (11%) | Unclear | 8 hours | Unclear | Medical record, fatal events: unclear | 3% |
| Nichols, 2009 [61] | 0%^c^ (11%) | 0% | Unclear | Unclear | Medical record, fatal events: unclear | Unclear |
| Nilsson, 2007 [62] | 0%^c^ (6%) | 0% | Overnight | Unclear | Hospital discharge diagnoses | Unclear |
| Preiss, 2010 [63] | 0%^c^ (7%) | Unclear | Unclear | Unclear | Hospital discharge diagnoses, death certificates | Unclear |
| Sarwar, 2010 [64] | 0%^c^ (7%) | 0% | 8 hours | Unclear | Central registries, death certificates | 1% |
| Sattar, 2008 [65] | 0%^c^ (7%) | 0% | Unclear | Unclear | Review board, death certificates | Unclear |
| Schillaci, 2004 [66] | 6%^c^ (8%) | 0% | Unclear | Unclear | Medical records, fatal events: unclear | 1% |
| Selvin, 2010 [67] | 0% (11%) | Unclear | 12 hours | Unclear | Medical records, death certificates | Unclear |
| Shin, 2009 [68] | 6% (9%) | 0% | 10 hours | 0% | Death certificates | 0% |
| Simons, 2000 [69] | 0%^c^ (8%) | 20% | 12 hours | Unclear | Register of hospitalizations, death certificates | 2% |
| Smith, 2002 [70] | 2% (11%) | Unclear | 8 hours | 5% | Medical records, death certificates | Unclear |
| Tai, 2004 [71] | 0%^c^ (11%) | 0% | 10 hours | Unclear | Myocardial register, hospital discharge register, death certificates | Unclear |
| Thomas, 2007 [72] | Unclear (9%) | 0% | 12 hours | Unclear | Death certificates | Unclear |
| Tsai, 2008 [73] | Unclear (10%) | 0% | Unclear | 3% | Death certificates | Unclear |
| Wang, 2007 [74] | 0%^c^ (9%) | 0% | Overnight | 37% | Medical records (only non-fatal events) | 42% |
| Watanabe, 2008 [75] | 12% (11%) | 0% | Overnight | Unclear | Elektrocardiograms at follow-up examination (only non-fatal events) | Unclear |
| Wilson, 2005 [76] | 0%^c^ (11%) | Unclear | Unclear | Unclear | Clinical examinations, physician outpatient record, hospitalizations | Unclear |
| Yarnell, 1998 [77] | 0% (7%) | Unclear | Overnight | 5% | Self-reports, electrocardiograms, hospital activity analysis, death certificates | 6% |
| Zhang, 2009 [78] | Unclear (9%) | 0% | Unclear | 17% | Unclear | Unclear |
| **Insulin** |  |  |  |  |  |  |
| Bonora, 2007 [79] | 7%^c^ (8%) | 0% | Overnight | 31% | Medical records, death certificates | 0% |
| Chien, 2008 [80] | 7% (10%) | 0% | 12 hours | Unclear | Self-reports and medical records, fatal events: unclear | Unclear |
| Dekker, 2005 [42] | 0%^c^ (7%) | Unclear | Overnight | 3% | Medical records, fatal events: unclear | 25% |
| Folsom, 1997 [81] | 0%^c^ (11%) | 0% | 12 hours | 15% | Hospital discharge diagnoses, electrocardiograms at follow-up examination, death certificates, relatives, interviews physicians | Unclear |
| Jeppesen, 2010 [82] | 0%^c^ (8%) | 0% | Overnight | 1% | Death certificates | Unclear |
| Juutilianen, 2006 [54] | 0% (9%) | 0% | 12 hours | Unclear | Death certificates | Unclear |
| Liu, 1992 [83] | 0%^c^ (11%) | 0% | Unclear | 37% | Electrocardiograms at follow-up examination (only non-fatal events) | Unclear |
| Nakamura, 2010 [84] | 0%^c^ (11%) | Unclear | Unclear | 1% | Medical records, fatal events: unclear | 1% |
| Nilsson, 2003 [85] | 0%^c^ (6%) | 0% | Unclear | 10% | Malmø heart register, death certificates | Unclear |
| Orchard, 1994 [86] | 0% (11%) | 0% | Unclear | 6% | Medical records, electrocardiograms at follow-up examination, death certificates | Unclear |
| Oterdoom, 2009 [87] | 0%^c^ (7%) | Unclear | 8 hours | 2% | Hospital discharge diagnosis, death certificates | Unclear |
| Pyorala, 1998 [88] | 0%^d^ (9%) | 0% | 12 hours | 2% | Medical records, death certificates, autopsy reports | 0% |
| Rutter, 2005 [89] | 0%^c^ (11%) | 0% | Overnight | 3% | Review board | Unclear |
| St-Pierre, 2005 [90] | 0% (11%) | 0% | 12 hours | Unclear | Death certificates, non-fatal events: unclear | 0% |
| Wang, 2007 [74] | 0%^c^ (9%) | 0% | Overnight | 37% | Medical records (only non-fatal events) | 42% |
| Yarnell, 1998 [77] | 0% (7%) | Unclear | Overnight | 5% | Self-reports, electrocardiograms, hospital activity analysis, death certificates | 6% |
| **HOMA-IR** |  |  |  |  |  |  |
| Arnlov, 2010 [91] | 0%^c^ (6%) | Unclear | Overnight | Unclear | Hospital discharge register, medial records for heart failure diagnosis, death certificates | 0% |
| Barr, 2010 [92] | 0%^c^ (8%) | Unclear | 9 hours | 1% | Medical records, death certificates | 19% |
| Bonora, 2007 [79] | 0%^c^ (8%) | 0% | Overnight | 31% | Medical records, death certificates | 0% |
| Chien, 2008 [80] | 7% (10%) | 0% | 12 hours | Unclear | Self-reports, medical records, fatal events: unclear | Unclear |
| Dekker, 2005 [42] | 0%^c^ (7%) | Unclear | Overnight | 3% | Medical records, fatal events: unclear | 25% |
| Hanley, 2002 [93] | 0% (11%) | Unclear | 12 hours | Unclear | Self-reports, death certificates | 35% |
| Hedblad, 2002 [94] | 0% (6%) | 0% | Overnight | 4% | Malmø Heart register, death certificates | Unclear |
| Hwang, 2009 [52] | 7%^c^ (9%) | Unclear | Overnight | 30% | Self-reports, medical records, fatal events: unclear | Unclear |
| Isomaa, 2001 [95] | Unclear (9%) | Unclear | Unclear | Unclear | Death certificates | 20% |
| Jeppesen, 2010 [82] | 0% (8%) | 0% | Overnight | 1% | Death certificates | Unclear |
| Nakamura, 2010 [84] | 0% (11%) | Unclear | Unclear | 1% | Medical records: fatal events: unclear | 1% |
| Nilsson, 2007 [62] | 0% (6%) | 0% | Overnight | Unclear | Hospital discharge diagnosis | Unclear |
| Onat, 2006 [96] | 0%^d^ (7%) | Unclear | 11 hours | 6% | Self-reports, electrocardiograms, fatal events: unclear | Unclear |
| Oterdoom, 2009 [87] | 0% (7%) | Unclear | 8 hours | 2% | Hospital discharge diagnoses, death certificates | Unclear |
| Resnick, 2003 [97] | 0% (11%) | Unclear | Unclear | 4% | Medical records, hospital discharge diagnosis | Unclear |
| Rundek, 2010 [98] | 0% (11%) | 0% | 12 hours | 44% | Death certificates, medical records | Unclear |
| Rutter, 2005 [89] | 0% (11%) | 0% | Overnight | Unclear | Review board | Unclear |
