# Supplementary material for: Insulin Resistance and Risk of Incident Cardiovascular Events in Adults without Diabetes: Meta-Analysis
Source: PLoS One. 2012 Dec 28;7(12):e52036. doi: 10.1371/journal.pone.0052036 (PMC3532497; doi:10.1371/journal.pone.0052036)
Supplement: References S1 — References of the included studies. (DOC) [file pone.0052036.s006.doc]

# Baba T, Amasaki Y, Soda M, Hida A, Imaizumi M et al. (2007) Fatty liver and uric acid levels predict incident coronary heart disease but not stroke among atomic bomb survivors in Nagasaki. Hypertens Res 30: 823-829.

# Balkau B, Shipley M, Jarrett RJ, Pyorala K, Pyorala M et al. (1998) High blood glucose concentration is a risk factor for mortality in middle-aged nondiabetic men. 20-year follow-up in the Whitehall Study, the Paris Prospective Study, and the Helsinki Policemen Study. Diabetes Care 21: 360-367.

# Barrett-Connor E, Wingard DL, Criqui MH, Suarez L (1984) Is borderline fasting hyperglycemia a risk factor for cardiovascular death? Journal of Chronic Diseases 37: 773-779.

# Bjornholt JV, Erikssen G, Aaser E, Sandvik L, Nitter-Hauge S et al. (1999) Fasting blood glucose: an underestimated risk factor for cardiovascular death. Results from a 22-year follow-up of healthy nondiabetic men. Diabetes Care 22: 45-49.

# Brunner EJ, Shipley MJ, Marmot MG, Kivimaki M, Witte DR (2010) Do the Joint British Society (JBS2) guidelines on prevention of cardiovascular disease with respect to plasma glucose improve risk stratification in the general population? Prospective cohort study. Diabet Med 27: 550-555.

# Cederberg H, Saukkonen T, Laakso M, Jokelainen J, Harkonen P et al. (2010) Postchallenge glucose, A1C, and fasting glucose as predictors of type 2 diabetes and cardiovascular disease: a 10-year prospective cohort study. Diabetes Care 33: 2077-2083.

# Chien KL, Lee BC, Lin HJ, Hsu HC, Chen MF (2009) Association of fasting and post-prandial hyperglycemia on the risk of cardiovascular and all-cause death among non-diabetic Chinese. Diabetes Research & Clinical Practice 83: e47-e50.

# Dekker JM, Girman C, Rhodes T, Nijpels G, Stehouwer CD et al. (2005) Metabolic syndrome and 10-year cardiovascular disease risk in the Hoorn Study. Circulation 112: 666-673.

# Doi Y, Ninomiya T, Hata J, Fukuhara M, Yonemoto K et al. (2010) Impact of glucose tolerance status on development of ischemic stroke and coronary heart disease in a general Japanese population: the Hisayama study. Stroke 41: 203-209.

# Eberly LE, Prineas R, Cohen JD, Vazquez G, Zhi X et al. (2006) Metabolic syndrome: risk factor distribution and 18-year mortality in the multiple risk factor intervention trial. Diabetes Care 29: 123-130.

# Ford ES (2004) The metabolic syndrome and mortality from cardiovascular disease and all-causes: findings from the National Health and Nutrition Examination Survey II Mortality Study. Atherosclerosis 173: 309-314.

# Girman CJ, Rhodes T, Mercuri M, Pyorala K, Kjekshus J et al. (2004) The metabolic syndrome and risk of major coronary events in the Scandinavian Simvastatin Survival Study (4S) and the Air Force/Texas Coronary Atherosclerosis Prevention Study (AFCAPS/TexCAPS). Am J Cardiol 93: 136-141.

# Hailpern SM, Cohen HW, Alderman MH (2006) Renal dysfunction predicts attenuation of ischemic heart disease mortality risk from elevated glucose among treated hypertensive patients. American Journal of Hypertension 19: 998-1004.

# Henry P, Thomas F, Benetos A, Guize L (2002) Impaired fasting glucose, blood pressure and cardiovascular disease mortality. Hypertension 40: 458-463.

# Ho JS, Cannaday JJ, Barlow CE, Mitchell TL, Cooper KH et al. (2008) Relation of the number of metabolic syndrome risk factors with all-cause and cardiovascular mortality. Am J Cardiol 102: 689-692.

# Hsu PF, Chuang SY, Cheng HM, Tsai ST, Chou P et al. (2008) Clinical significance of the metabolic syndrome in the absence of established hypertension and diabetes: A community-based study. Diabetes Research & Clinical Practice 79: 461-467.

# Hunt KJ, Resendez RG, Williams K, Haffner SM, Stern MP et al. (2004) National Cholesterol Education Program versus World Health Organization metabolic syndrome in relation to all-cause and cardiovascular mortality in the San Antonio Heart Study. Circulation 110: 1251-1257.

# Hwang YC, Jee JH, Oh EY, Choi YH, Lee MS et al. (2009) Metabolic syndrome as a predictor of cardiovascular diseases and type 2 diabetes in Koreans. Int J Cardiol 134: 313-321.

# Jeppesen J, Hansen TW, Rasmussen S, Ibsen H, Torp-Pedersen C et al. (2007) Insulin Resistance, the Metabolic Syndrome, and Risk of Incident Cardiovascular Disease: A Population-Based Study. Journal of the American College of Cardiology 49: 2112-2119.

# Juutilainen A, Lehto S, Ronnemaa T, Pyorala K, Laakso M (2006) Proteinuria and metabolic syndrome as predictors of cardiovascular death in non-diabetic and type 2 diabetic men and women. Diabetologia 49: 56-65.

# Khang YH, Cho SI, Kim HR (2010) Risks for cardiovascular disease, stroke, ischaemic heart disease, and diabetes mellitus associated with the metabolic syndrome using the new harmonised definition: findings from nationally representative longitudinal data from an Asian population. Atherosclerosis 213: 579-585.

# Kokubo Y, Okamura T, Watanabe M, Higashiyama A, Ono Y et al. (2010) The combined impact of blood pressure category and glucose abnormality on the incidence of cardiovascular diseases in a Japanese urban cohort: the Suita Study. Hypertens Res 33: 1238-1243.

# Lapidus L, Bengtsson C, Blohme G, Lindquist O, Nystrom E (1985) Blood glucose, glucose tolerance and manifest diabetes in relation to cardiovascular disease and death in women. A 12-year follow-up of participants in the population study of women in Gothenburg, Sweden. Acta Med Scand 218: 455-462.

# Liu J, Grundy SM, Wang W, Smith SC, Jr., Vega GL et al. (2007) Ten-year risk of cardiovascular incidence related to diabetes, prediabetes, and the metabolic syndrome. American Heart Journal 153: 552-558.

# Marin A, Medrano MJ, Gonzalez J, Pintado H, Compaired V et al. (2006) Risk of ischaemic heart disease and acute myocardial infarction in a Spanish population: observational prospective study in a primary-care setting. BMC Public Health 6:38.

# Nakanishi N, Takatorige T, Fukuda H, Shirai K, Li W et al. (2004) Components of the metabolic syndrome as predictors of cardiovascular disease and type 2 diabetes in middle-aged Japanese men. Diabetes Research & Clinical Practice 64: 59-70.

# Nichols GA, Koro CE, Kolatkar NS (2009) The incidence of heart failure among nondiabetic patients with and without impaired fasting glucose. J Diabetes Complications 23: 224-228.

# Nilsson PM, Engstrom G, Hedblad B (2007) The metabolic syndrome and incidence of cardiovascular disease in non-diabetic subjects--a population-based study comparing three different definitions. Diabetic Medicine 24: 464-472.

# Preiss D, Welsh P, Murray HM, Shepherd J, Packard C et al. (2010) Fasting plasma glucose in non-diabetic participants and the risk for incident cardiovascular events, diabetes, and mortality: results from WOSCOPS 15-year follow-up. Eur Heart J 31: 1230-1236.

# Sarwar N, Aspelund T, Eiriksdottir G, Gobin R, Seshasai SR et al. (2010) Markers of dysglycaemia and risk of coronary heart disease in people without diabetes: Reykjavik prospective study and systematic review. PLoS Medicine 7: e1000278.

# Sattar N, McConnachie A, Shaper AG, Blauw GJ, Buckley BM et al. (2008) Can metabolic syndrome usefully predict cardiovascular disease and diabetes? Outcome data from two prospective studies. Lancet 371: 1927-1935.

# Schillaci G, Pirro M, Vaudo G, Gemelli F, Marchesi S et al. (2004) Prognostic value of the metabolic syndrome in essential hypertension. Journal of the American College of Cardiology 43: 1817-1822.

# Selvin E, Steffes MW, Zhu H, Matsushita K, Wagenknecht L et al. (2010) Glycated hemoglobin, diabetes, and cardiovascular risk in nondiabetic adults. N Engl J Med 362: 800-811.

# Shin CY, Yun KE, Park HS (2009) Blood pressure has a greater impact on cardiovascular mortality than other components of metabolic syndrome in Koreans. Atherosclerosis 205: 614-619.

# Simons LA, Friedlander Y, McCallum J, Simons J (2000) Fasting plasma glucose in non-diabetic elderly women predicts increased all-causes mortality and coronary heart disease risk. Australian & New Zealand Journal of Medicine 30: 41-47.

# Smith NL, Barzilay JI, Shaffer D, Savage PJ, Heckbert SR et al. (2002) Fasting and 2-hour postchallenge serum glucose measures and risk of incident cardiovascular events in the elderly: the Cardiovascular Health Study. Arch Intern Med 162: 209-216.

# Tai ES, Goh SY, Lee JJ, Wong MS, Heng D et al. (2004) Lowering the criterion for impaired fasting glucose: impact on disease prevalence and associated risk of diabetes and ischemic heart disease. Diabetes Care 27: 1728-1734.

# Thomas GN, Schooling CM, McGhee SM, Ho SY, Cheung BM et al. (2007) Metabolic syndrome increases all-cause and vascular mortality: the Hong Kong Cardiovascular Risk Factor Study. Clin Endocrinol (Oxf) 66: 666-671.

# Tsai SP, Wen CP, Chan HT, Chiang PH, Tsai MK et al. (2008) The effects of pre-disease risk factors within metabolic syndrome on all-cause and cardiovascular disease mortality. Diabetes Research & Clinical Practice 82: 148-156.

# Wang JJ, Li HB, Kinnunen L, Hu G, Jarvinen TM et al. (2007) How well does the metabolic syndrome defined by five definitions predict incident diabetes and incident coronary heart disease in a Chinese population? Atherosclerosis 192: 161-168.

# Watanabe H, Tanabe N, Watanabe T, Darbar D, Roden DM et al. (2008) Metabolic syndrome and risk of development of atrial fibrillation: the Niigata preventive medicine study. Circulation 117: 1255-1260.

# Wilson PW, D'Agostino RB, Parise H, Sullivan L, Meigs JB (2005) Metabolic syndrome as a precursor of cardiovascular disease and type 2 diabetes mellitus. Circulation 112: 3066-3072.

# Yarnell JWG, Patterson CC, Bainton D, Sweetnam PM (1998) Is metabolic syndrome a discrete entity in the general populaton? Evidence from the caerphilly and speedwell population studies. Heart 79: 248-252.

# Zhang WW, Liu CY, Wang YJ, Xu ZQ, Chen Y et al. (2009) Metabolic syndrome increases the risk of stroke: a 5-year follow-up study in a Chinese population. Journal of Neurology 256: 1493-1499.

# Bonora E, Kiechl S, Willeit J, Oberhollenzer F, Egger G et al. (2007) Insulin resistance as estimated by homeostasis model assessment predicts incident symptomatic cardiovascular disease in caucasian subjects from the general population: the Bruneck study. Diabetes Care 30: 318-324.

# Chien KL, Hsu HC, Su TC, Chen MF, Lee YT et al. (2008) Fasting and postchallenge hyperglycemia and risk of cardiovascular disease in Chinese: The Chin-Shan Community Cardiovascular Cohort study. American Heart Journal 156: 996-1002.

# Folsom AR, Szklo M, Stevens J, Liao F, Smith R et al. (1997) A prospective study of coronary heart disease in relation to fasting insulin, glucose, and diabetes. The Atherosclerosis Risk in Communities (ARIC) Study. Diabetes Care 20: 935-942.

# Jeppesen J, Hansen TW, Torp-Pedersen C, Madsbad S, Ibsen H et al. (2010) Relationship Between Common Lipoprotein Lipase Gene Sequence Variants, Hyperinsulinemia, and Risk of Ischemic Heart Disease: a Population-Based Study. Atherosclerosis 211:506-511.

# Liu QZ, Knowler WC, Nelson RG, Saad MF, Charles MA et al. (1992) Insulin treatment, endogenous insulin concentration, and ECG abnormalities in diabetic Pima Indians. Cross-sectional and prospective analyses. Diabetes 41: 1141-1150.

# Nakamura K, Sakurai M, Miura K, Morikawa Y, Ishizaki M et al. (2010) Homeostasis model assessment of insulin resistance and the risk of cardiovascular events in middle-aged non-diabetic Japanese men. Diabetologia 53: 1894-1902.

# Nilsson P, Nilsson JA, Hedblad B, Eriksson KF, Berglund G (2003) Hyperinsulinaemia as long-term predictor of death and ischaemic heart disease in nondiabetic men: The Malmo Preventive Project. Journal of Internal Medicine 253: 136-145.

# Orchard TJ, Eichner J, Kuller LH, Becker DJ, McCallum LM et al. (1994) Insulin as a predictor of coronary heart disease: interaction with apolipoprotein E phenotype. A report from the Multiple Risk Factor Intervention Trial. Ann Epidemiol 4: 40-45.

# Oterdoom LH, de Vries AP, Gansevoort RT, de Jong PE, Gans RO et al. (2009) Fasting insulin is a stronger cardiovascular risk factor in women than in men. Atherosclerosis 203: 640-646.

# Pyorala M, Miettinen H, Laakso M, Pyorala K (1998) Hyperinsulinemia and the risk of stroke in healthy middle-aged men: The 22-year follow-up results of the Helsinki Policemen Study. Stroke 29: 1860-1866.

# Rutter MK, Meigs JB, Sullivan LM, D'Agostino RB, Sr., Wilson PW (2005) Insulin resistance, the metabolic syndrome, and incident cardiovascular events in the Framingham Offspring Study. Diabetes 54: 3252-3257.

# St-Pierre AC, Cantin B, Mauriege P, Bergeron J, Dagenais GR et al. (2005) Insulin resistance syndrome, body mass index and the risk of ischemic heart disease. CMAJ Canadian Medical Association Journal 172: 1301-1305.

# Arnlov J, Ingelsson E, Sundstrom J, Lind L (2010) Impact of body mass index and the metabolic syndrome on the risk of cardiovascular disease and death in middle-aged men. Circulation 121: 230-236.

# Barr EL, Cameron AJ, Balkau B, Zimmet PZ, Welborn TA et al. (2010) HOMA insulin sensitivity index and the risk of all-cause mortality and cardiovascular disease events in the general population: the Australian Diabetes, Obesity and Lifestyle Study (AusDiab) study. Diabetologia 53: 79-88.

# Hanley AJ, Williams K, Stern MP, Haffner SM (2002) Homeostasis model assessment of insulin resistance in relation to the incidence of cardiovascular disease: the San Antonio Heart Study. Diabetes Care 25: 1177-1184.

# Hedblad B, Nilsson P, Engstrom G, Berglund G, Janzon L (2002) Insulin resistance in non-diabetic subjects is associated with increased incidence of myocardial infarction and death. Diabetic Medicine 19: 470-475.

# Isomaa B, Almgren P, Tuomi T, Forsen B, Lahti K et al. (2001) Cardiovascular morbidity and mortality associated with the metabolic syndrome. Diabetes Care 24: 683-689.

# Onat A, Hergenc G, Turkmen S, Yazici M, Sari I et al. (2006) Discordance between insulin resistance and metabolic syndrome: features and associated cardiovascular risk in adults with normal glucose regulation. Metabolism: Clinical & Experimental 55: 445-452.

# Resnick HE, Jones K, Ruotolo G, Jain AK, Henderson J et al. (2003) Insulin resistance, the metabolic syndrome, and risk of incident cardiovascular disease in nondiabetic american indians: the Strong Heart Study. Diabetes Care 26: 861-867.

1. Rundek T, Gardener H, Xu Q, Goldberg RB, Wright CB et al. (2010) Insulin resistance and risk of ischemic stroke among nondiabetic individuals from the northern Manhattan study. Archives of Neurology 67: 1195-1200.
